# Carbazole‐ and Fluorene‐Fused Aza‐BODIPYs: NIR Fluorophores with High Brightness and Photostability

**DOI:** 10.1002/chem.202100965

**Published:** 2021-06-02

**Authors:** Tanja Rappitsch, Sergey M. Borisov

**Affiliations:** ^1^ Graz University of Technology Institute of Analytical Chemistry and Food Chemistry Stremayrgasse 9 8010 Graz Austria

**Keywords:** dyes, fluorescence, near-infrared, pH, sensing

## Abstract

Three new aza‐BODIPY dyes incorporating fused fluorene or carbazole moieties have been prepared. The dyes show significant enhancement of photophysical properties compared to the parent 1,3,5,7‐tetraphenyl aza‐BODIPY (TPAB): a bathochromic shift of the absorption maximum (up to 2700 cm^−1^) and emission maximum (up to 2270 cm^−1^); an almost threefold increase in molar absorption coefficients (to ca. 230 000 M^−1^ cm^−1^) and a significant increase in the fluorescence quantum yield to 49–66 %. Owing to the combination of these properties, the new aza‐BODIPY dyes belong to the brightest NIR dyes reported. The dyes also show excellent photostability. Due to their outstanding properties, the new dyes represent a promising platform for further exploration in biomedical research. A pH indicator containing only one fused carbazole unit was also prepared and shows absorption and emission spectra that are bathochromically shifted by about 110 and 100 nm, respectively, compared to the indicator dye based on the TPAB chromophore.

## Graphical abstract

**Look on the bright side**: Three new fluorescent aza‐BODIPY dyes fused with fluorene or carbazole substituents show strong bathochromic shifts (up to 139 nm) of absorption and emission maxima compared to the parent 1,3,5,7‐tetraphenyl aza‐BODIPY (TPAB) and are highly photostable. Two of the dyes show excellent fluorescence brightness due to exceptionally high molar absorption coefficients (ca. 230 000 M^−1^ cm^−1^) and emission quantum yields (up to 0.66); the third dye was rendered pH sensitive by introducing a chlorophenol motif.

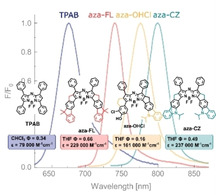

## Introduction

Near‐infrared (NIR) dyes are of particular interest in biomedical research.[^1^, ^2^] Compared to visible light, NIR light has a deeper penetration depth through body tissue and also generates far less autofluorescence of biomolecules ensuring low background noise and high signal to noise ratio.^[3]^ The resulting high resolution is especially desirable in fluorescence imaging^[4]^ and photoacoustic imaging.^[5]^ NIR dyes with high probability of intersystem crossing are of interest for photodynamic therapy.^[6]^ The advancement of these methods triggers the design of new NIR dyes with optimized spectral properties in the NIR spectral range.

Typical NIR emitting dyes belong to the group of squaraines,[^7^, ^8^] cyanines,[^9^, ^10^] boron dipyrromethenes (BODIPYs)[^11^, ^12^, ^13^] or modified rhodamines[^14^, ^15^] (Table S2 in the Supporting Information). Some limitations of these dyes include a tendency to accumulate in mitochondria and aggregate in the case of rhodamines,[^16^, ^17^] susceptibility to chemical attack by strong nucleophiles in the case of squaraines,^[18]^ moderate quantum yields (<0.3) and poor photostability of cyanines. Among cyanine dyes, indocyanine green is the only US Food and Drug Administration (FDA)‐approved cyanine probe for in vivo use in medical applications but the photostability and the chemical properties for this dye are inadequate.[^19^, ^20^, ^21^]

**Figure 1:**
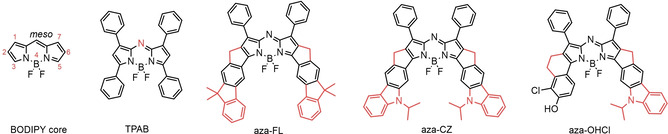
Structure of the BODIPY core, tetraphenyl aza‐BODPY (TPAB) and aza‐BODIPYs aza‐FL, aza‐CZ and aza‐OHCl (reported in this work).

Similar to cyanines, BODIPY dyes have high absorption coefficients and possess narrow absorption‐ and emission bands (FWHM_abs_ around 700 cm^−1^) but are superior in terms of fluorescence quantum yields and photostability. Unfortunately, most of the BODIPY dyes emit below 600 nm.[^22^, ^23^, ^24^] Structurally related aza‐BODIPY dyes (most common as 1,3,5,7‐tetraaryl‐substituted derivatives such as TPAB; Figure 1) possess a nitrogen atom in the *meso‐*position.[^25^, ^26^, ^27^] Compared to BODIPYs, these dyes are characterised by bathochromically shifted absorption and emission spectra (maxima in red and far‐red region, respectively) but also by significantly broader bands (FWHM_abs_ around 1200 cm^−1^) and lower fluorescence quantum yields (*ϕ*_F_<0.36).[^28^, ^29^] Common strategies to obtain far‐red and NIR absorbing aza‐BODIPYs include: i) introduction of donor groups[^28^, ^30^, ^31^, ^32^, ^33^] and ii) π‐extension with large aromatic substituents that are either only partly conjugated[^34^, ^35^, ^36^, ^37^, ^38^] or fully condensed.[^39^, ^40^, ^41^, ^42^, ^43^] Unfortunately, a bathochromic shift of about 80 nm compared to the parent TPAB is usually accompanied by a decrease of the quantum yield (<0.3; Table S3). We recently showed that the absorption and emission bands of BODIPY dyes can be strongly modulated via extension of the π‐system with conjugated fluorene or carbazole motifs.^[44]^ Importantly, the modified dyes retained the excellent photophysical properties of the parent BODIPY chromophore. Therefore, we considered it of significant interest to investigate if the similar substitution strategy is also efficient in the case of the aza‐BODIPY dyes and can yield novel bright NIR emitting compounds of these dye class.

In this contribution, we present new bright NIR aza‐BODIPY dyes incorporating rigid fused carbazole and fluorene moieties (Figure 1). The new aza‐BODIPYs offer outstanding photophysical properties and are among the brightest NIR emitting aza‐BODIPYs reported (Table S3). In this study we also investigated the possibility to introduce a receptor group into the structure of one aza‐BODIPY to make them pH‐sensitive.

## Results and Discussion

### Synthesis

**Scheme 1:**
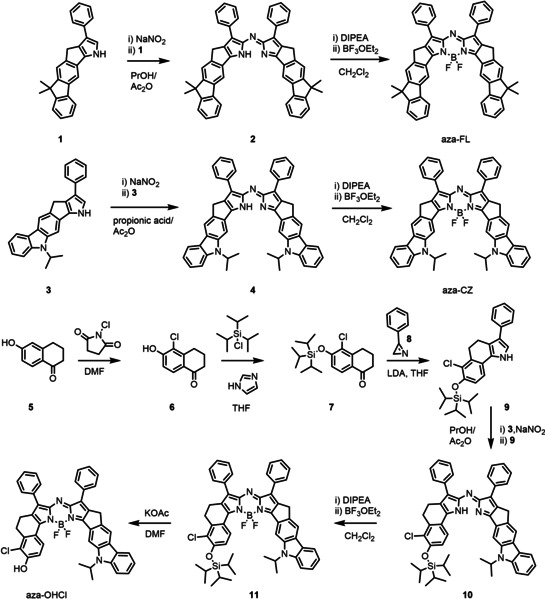
Synthesis of the aza‐BODIPY dyes.

The synthesis of aza‐BODIPYs aza‐FL, aza‐CZ and aza‐OHCl is shown in Scheme 1. The aza‐BODIPYs were prepared according to a reported method that relies on reaction of pyrrole with a nitrosopyrrole, followed by complexation with BF_3_OEt_2_ in presence of *N*,*N*‐diisopropylethylamine (DIPEA).^[25]^ Both symmetric (aza‐FL, aza‐CZ) and asymmetric (aza‐BODIPY **11**) aza‐BODIPYs are synthesized by using this method. Pyrroles **1** and **3** were prepared according to previously published method.^[44]^ The pyrrole building block for the aza‐BODIPY derivative aza‐OHCl is synthesized in a 3 step route starting from 6‐hydroxy‐1‐tetralone **5**. Chlorination using *N*‐chlorosuccinimide and protection of the alcohol moiety with triisopropylchlorosilane gives ketone **7**. The tetralone‐fused pyrrole **9** is obtained in 47 % yield in reaction between ketone **7** and 3‐phenyl‐2*H*‐azirene in presence of lithium diisopropylamide. Condensation of **9** with the nitrosylated pyrrole **3** and subsequent chelation gives the protected aza‐BODIPY dye **11** that is converted to aza‐OHCl in 79 % yield in a deprotection step using KOAc in DMF.

### Photophysical properties

**Figure 2:**
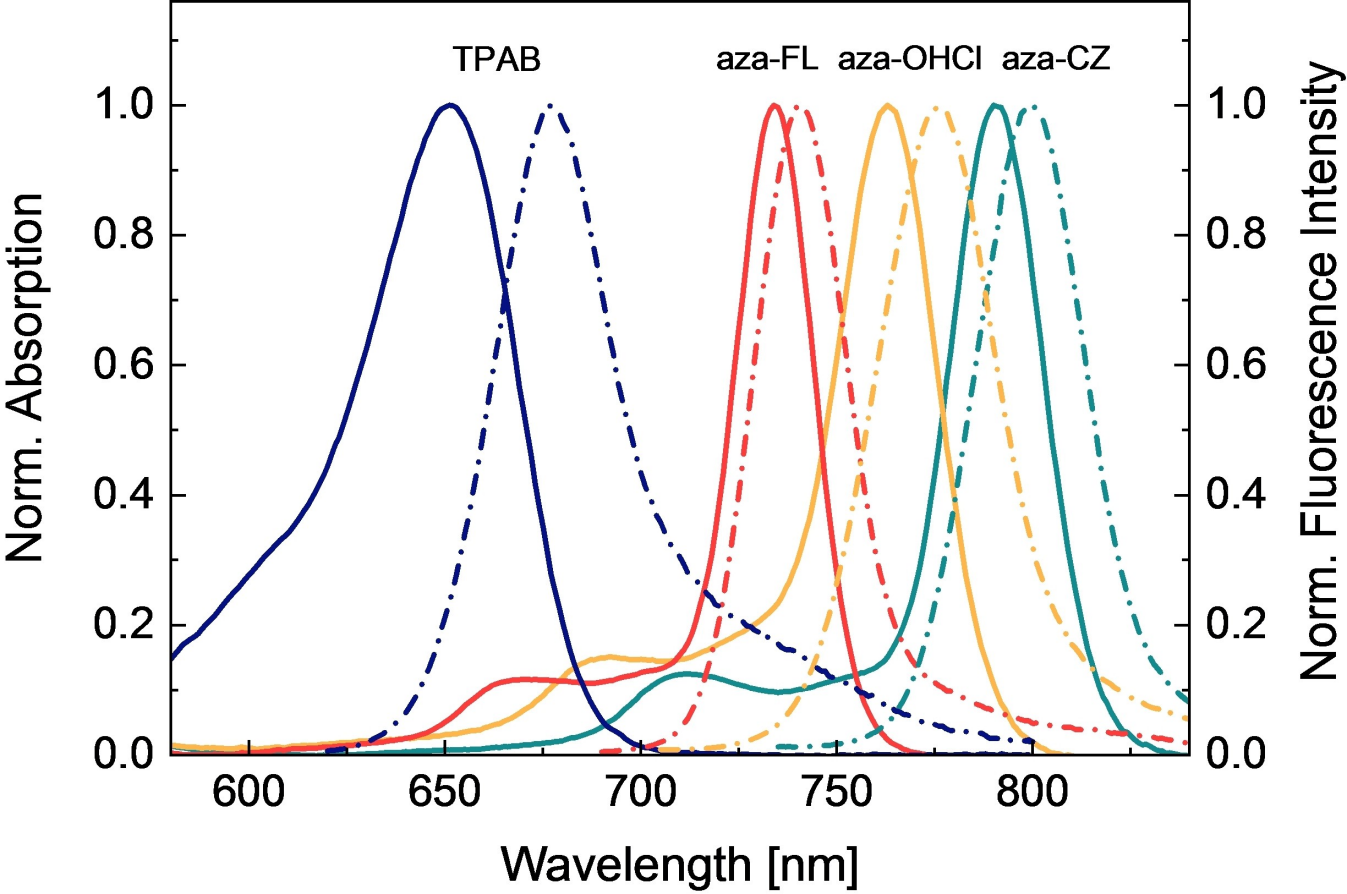
Normalized absorption (solid lines) and emission (dashed lines) spectra of dyes TPAB (blue), aza‐FL (red), aza‐OHCl (yellow), and aza‐CZ (turquoise) in THF.

The spectroscopic properties of the new aza‐BODIPYs aza‐FL, aza‐CZ and aza‐OHCl in tetrahydrofuran (THF) are compiled in Table 1. All the aza‐BODIPY dyes exhibit intense absorption bands between 734 and 791 nm (Figure 2) and weaker bands in the UV/Vis spectral range (Figure S1). In comparison to the simplest of the aza‐BODIPY family (1,3,5,7‐tetraphenyl aza‐BODIPY, TPAB) the absorption maximum shifts by 82 nm (1710 cm^−1^) for aza‐FL due to the substitution of the phenyl ring by fused fluorene moiety. A much stronger shift of 139 nm (2700 cm^−1^) is induced by introduction of electron‐rich carbazole group (aza‐CZ). For the asymmetrical aza‐BODIPY aza‐OHCl a bathochromic shift of 111 nm (2230 cm^−1^) can be seen. It is also evident that the absorption bands in the new aza‐BODIPYs are much narrower than for TPAB (Figure 2). In fact, FWHM_abs_ for aza‐FL, aza‐CZ and aza‐OHCl is 436, 448, and 522 cm^−1^, respectively, compared to 1135 cm^−1^ for TPAB. Simultaneously, the molar absorption coefficients are strongly enhanced reaching the values around 230 000 M^−1^ cm^−1^ for symmetric aza‐FL and aza‐CZ (Table 1).

**Table 1:** Photophysical properties of aza‐BODIPY dyes in THF at room temperature.

Dye	*λ*_abs_ [nm]	*λ*_em_ [nm]	*ϵ* [M^−1^ cm^−1^]^[a]^	*ϕ*_F_	τ [ns]
aza‐FL	734	741	229 000	0.66±0.04^[b]^	4.3
aza‐CZ	791	800	237 000	0.49±0.04^[c]^	2.3
aza‐OHCl^[e]^	763	776	161 000	0.16±0.01^[b]^	1.7
TPAB	652	677	79 000^[d]^	0.34^[d]^	0.9

[a] Error: within 5 % for all dyes. [b] Relative to HITCI (*ϕ*=0.283) in EtOH.^[45]^ [c] Relative to IR‐125 (*ϕ*=0.132) and IR‐140 in EtOH (*ϕ*=0.167).^[45]^ [d] Measured in chloroform.^[28]^ [e] for the protonated form (protonated with 1 v%/v trifluoracetic acid).

The trend observed in absorption maxima was the same for fluorescence emission maxima (Table 1). The Stokes shift for the dyes aza‐FL, aza‐CZ and aza‐OHCl is 129, 142 and 220 cm^−1^, respectively, that is much smaller than for the parent TPAB dye (Stokes shift of 566 cm^−1^). The fluorescence quantum yields for aza‐FL and aza‐CZ are exceptionally high (0.66 and 0.49, respectively) which is much higher than reported for TPAB (0.34 in chloroform).^[28]^ These values are even more impressive considering that the fluorescence quantum yields generally tend to decrease for dyes showing emission in far‐red and NIR part of the spectrum. Dye aza‐OHCl has somewhat lower quantum yield (0.16, protonated form) which can nevertheless be considered acceptable for the dye emitting in the NIR part of the electromagnetic spectrum. The fluorescence of the dyes in solution decays monoexponentially (Figure S2), and the lifetimes of the aza‐BODIPYs are in the range of 1.7–4.3 ns (Table 1) which is consistent with the values for aza‐BODIPYs reported.^[46]^ The photophysical properties of the new aza‐BODIPY dyes were also investigated in two other organic solvents: toluene and acetonitrile which polarity is lower and higher, respectively, than that of tetrahydrofuran. The absorption and the fluorescence spectra of aza‐FL, aza‐CZ, aza‐OHCl and TPAB shift bathochromically up to 10 nm as the solvent polarity decreases (Table S1). The fluorescence quantum yield and the decay times for aza‐FL are virtually not affected by the solvent polarity. In the case of aza‐CZ, aza‐OHCl and TPAB significant decrease of the fluorescence quantum yield and decay times is observed in rather polar acetonitrile. Some decrease of these parameters is already seeing on going from toluene to tetrahydrofuran.

### Photostability

**Figure 3:**
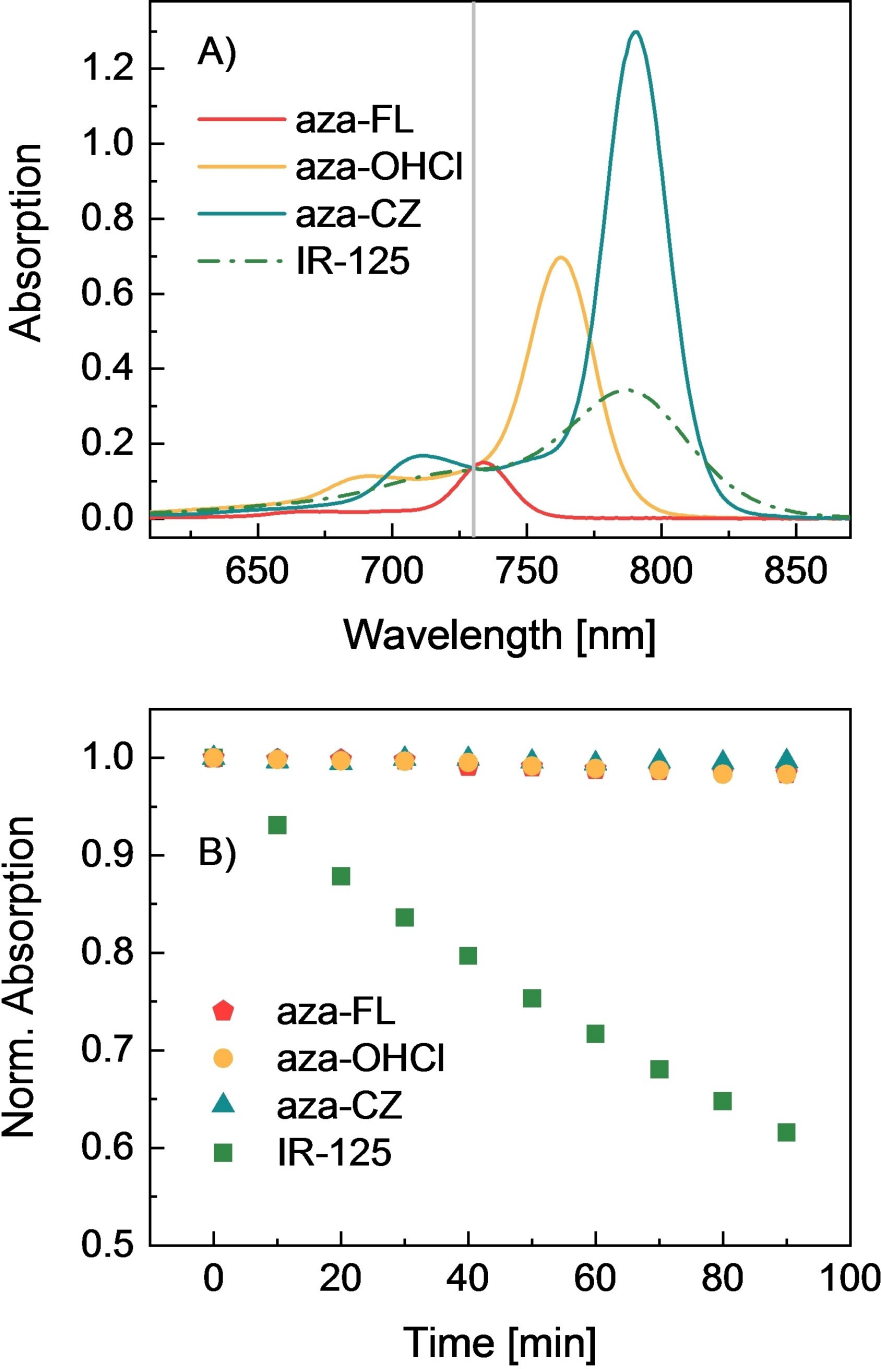
A) Absorption spectra of aza‐FL, aza‐CZ and aza‐OHCl compared to IR‐125 (the gray line indicates the excitation wavelength corresponding to the LED peak, 730 nm) B) Photodegradation of aza‐FL, aza‐CZ, aza‐OHCl (in THF) and IR‐125 (in EtOH) upon irradiation of the solutions with the far‐red light of a high‐power LED array (*λ*=730 nm, photon flux: ∼15 000 μmol s^−1^ m^−2^). The absorption spectra measured over the course of the photobleaching experiment are provided in Figures S3‐S6.

Photostability is a very important parameter for possible application in biomedicine to gain steady signal read‐out. Strong photobleaching makes quantitative analysis over time difficult and limits the accuracy of the detection. Indocyanine green (IR‐125) was chosen as the reference compound for photobleaching experiment because its spectral properties (*λ*_max, abs_=787 nm in EtOH; *λ*_max, em_=818 nm, Figure 3A) show strong similarity with those of aza‐CZ and because it has been previously employed in evaluation of the photostability of aza‐BODIPY dyes.^[30]^ Comparative photobleaching experiments for aza‐FL, aza‐CZ and aza‐OHCl and IR‐125 showed, that around 40 % of IR‐125 decomposed after 90 min irradiation with a high‐power far‐red LED array (*λ*=730 nm), Figure 3B. Under the same irradiation conditions, less than 2 % of the aza‐BODIPYs aza‐FL, aza‐CZ and aza‐OHCl bleached away, confirming their superior photostability (Figure 3B). Aza‐CZ appears to be the most photostable with barely detectable bleaching (0.5 % degradation registered after 90 min of continuous irradiation). In the same conditions, about 1.7 % of aza‐FL and aza‐OHCl degraded. It can be concluded that the π‐extended aza‐BODIPYs represent an attractive platform for future application in biomedicine, combining outstanding brightness and unmatched photostability with absorption and emission in the NIR region.

### Acid‐base equilibrium of aza‐OHCl

**Figure 4:**
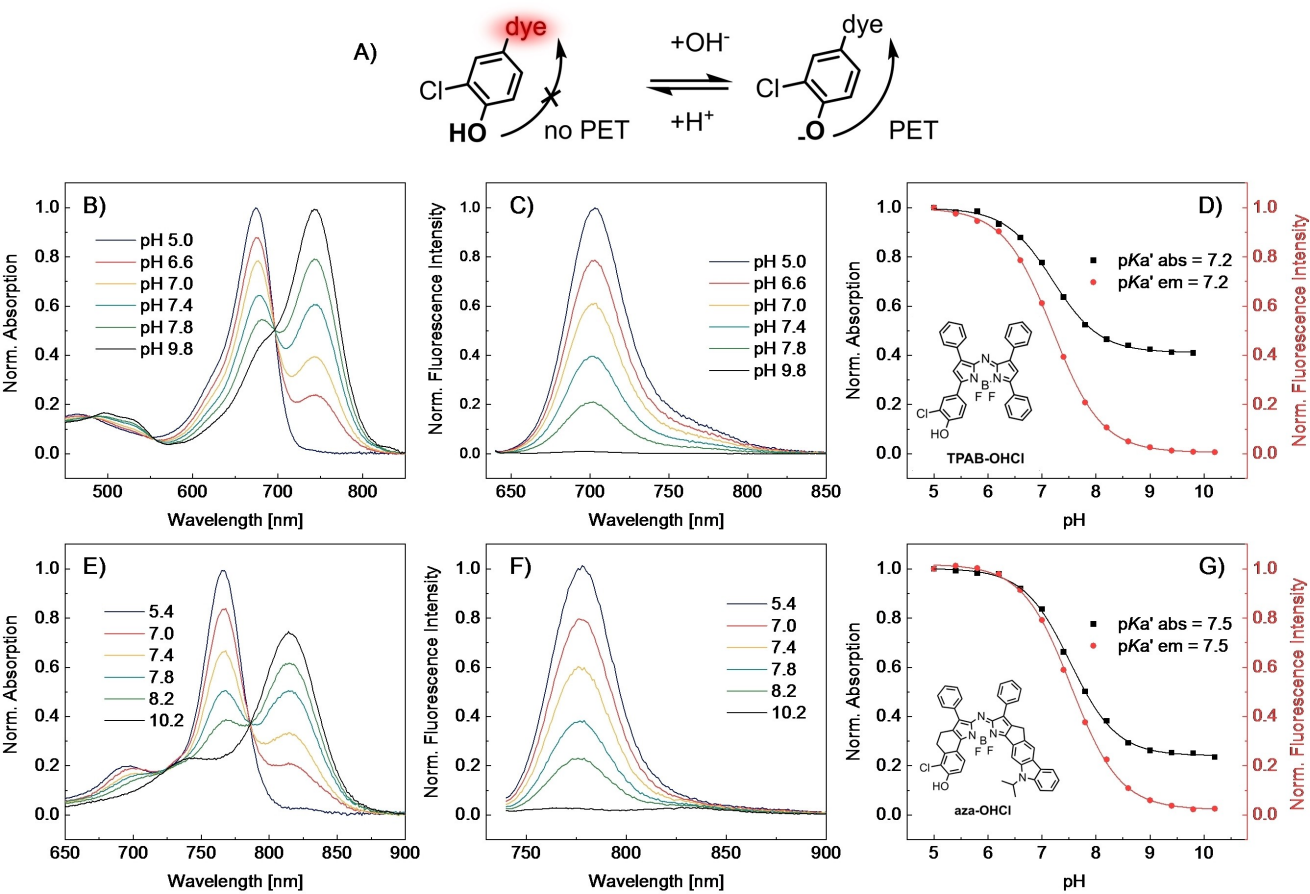
pH‐sensing properties of B)‐D) TPAB‐OHCl and E)‐G) aza‐OHCl. A) Principle of pH sensing by PET sensor. B) and E) pH dependency of the absorption spectra. C) pH dependency of the emission spectra of TPAB‐OHCl (*λ*_ex_=630 nm). F) pH dependency of the emission spectra of aza‐OHCl (*λ*_ex_=725 nm). D) and G) Corresponding response curves with fit using a Boltzmann sigmoid. All the spectra were acquired in THF/EtOH/aqueous buffer (1 : 1 : 1, *v*/*v*/*v*) at room temperature.

In contrast to new aza‐BODIPY dyes aza‐FL and aza‐CZ that are inert to environmental changes, aza‐OHCl contains a phenol receptor that renders the dye pH sensitive. Figure 4 shows pH dependency of the absorption and emission spectra for aza‐OHCl in THF/EtOH/aqueous buffer (1 : 1 : 1, *v*/*v*/*v*) in comparison with spectral properties of TPAB‐OHCl that bears the same receptor group. Similar to the non‐rigid analogue (Figure 4B), the absorption spectrum of aza‐OHCl (Figure 4E) shifts bathochromically upon deprotonation (by 69 and 49 nm for TPAB‐OHCl and aza‐OHCl, respectively). Note that the deprotonated form of the new indicator shows comparably little absorption in the absorption maximum of the neutral form whereas the neutral form does not show any absorption at all in the absorption maximum of the deprotonated form. This property makes the dye potentially suitable as absorptiometric pH indicator operating in the NIR spectral range.

As can also be seen (Figure 4C and F), fluorescence is completely “switched off” upon deprotonation of the OH group; this is attributed to efficient photoinduced electron transfer (Figure 4A).^[47]^ The response follows Boltzmann sigmoidal dependency (Figure 4D and G). The response of aza‐OHCl is thus similar to that of the previously reported TPAB‐OHCl aza‐BODIPY dye^[48]^ that possesses the same 3‐chloro‐4‐hydroxyphenyl receptor group. The apparent p*K*_a_ value of aza‐OHCl is slightly higher than for TPAB‐OHCl (7.5 and 7.2, respectively). This might be due to the electron‐donating character of the rigid C_2_H_4_ bridge or higher hydrophobicity of the dye that makes the formation of the charged form less favorable. Importantly, aza‐OHCl shows absorption and emission spectra that are bathochromically shifted by about 100 and 80 nm, respectively, compared to TPAB‐OHCl (Figure 4, Table 2). Another notable improvement is the approximately twofold higher molar absorption coefficients in the case of aza‐OHCl (Table 2). Such properties make the new dye potentially attractive for a number of applications in highly scattering media such as tissues. Apart from fluorescence imaging of pH, application of the dye as a photoacoustic (PA) imaging probe appears to be particularly interesting. This noninvasive imaging technique relies on combination of pulsed laser excitation and ultrasonic detection.^[49]^ Ideal PA probes should have strong NIR absorption and excellent photostability as well as comparably low quantum yields to generate a strong PA signal for reasonable signal read‐out. Previous studies demonstrated successful application of aza‐BODIPYs as PA probes.^[5]^ Miao et al. showed that TPAB‐OHCl is especially promising for PA imaging of pH distribution since “turn off” of fluorescence upon deprotonation of OH‐group of the receptor is associated with enhancement of PA signal.^[50]^ Due to significantly higher molar absorption coefficients and even better match with the NIR optical window the new indicator aza‐OHCl may be very attractive for such applications.

**Table 2:** Spectral and pH sensing properties of aza‐BODIPYs aza‐OHCl and TPAB‐OHCl in THF/EtOH/aqueous buffer (1 : 1 : 1, *v*/*v*/*v*) at room temperature.

Dye	*λ*_abs‐acid_/ *λ*_abs‐base_ [nm]	*λ*_em‐acid_ [nm]	p*K*_a’_ abs	p*K*_a’_ em	*ϵ* [M^−1^ cm^−1^]^[a]^	*ϕ*_F_^[a]^
aza‐OHCl	766/815	779	7.5	7.5	161 000	0.16
TPAB‐OHCl	674/743	703	7.2	7.2	80 600^[48]^	0.16^[b]^

[a] For the protonated form, measured in THF.

### Application as security inks

**Figure 5:**
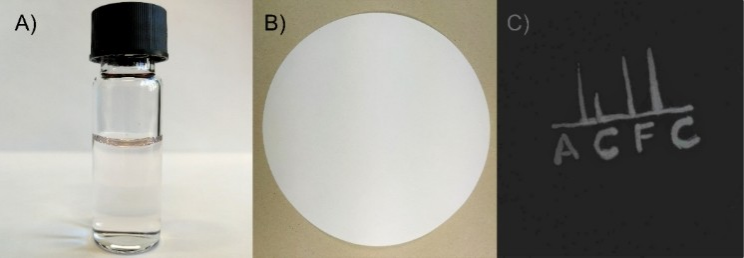
Security ink based on aza‐CZ immobilized into RL‐100 nanoparticles. A) Aqueous dispersion of nanoparticles under ambient light (*C*=0.15 mg/mL of beads). B) Filter paper with invisible writing under ambient light. C) Fluorescence image of the filter paper obtained with a NIR camera.

Luminescence is widely used in anti‐counterfeiting applications and there is still considerable interest in new organic and inorganic luminescent materials that can be applied as security inks.[^51^, ^52^, ^53^, ^54^, ^55^] The new aza‐BODIPYs may be attractive for these applications due to strong “invisible” NIR emission and high photostability which is essential for long‐term application of the dyes under ambient light conditions. A security ink was prepared by immobilizing aza‐CZ into water‐dispersible RL‐100 nanoparticles (∅ ca. 35 nm), a polymer on basis of poly(methyl methacrylate) that contains quaternary ammonium groups. The nanoprecipitation method is attractive for its simplicity and versatility since many other polymers can also be used.^[56]^ A nearly colorless aqueous dispersion of the nanoparticles (Figure 5A) was applied on a filter paper with the writing invisible under ambient light (Figure 5B). Figure 5C shows that the pattern becomes clearly visible when the paper is illuminated with a blue LED (*λ*=451 nm) and the emission is detected by a NIR camera. Note that although the most intense absorption band of aza‐CZ is located in the NIR part of the electromagnetic spectrum, the dye also shows weaker absorption in the UV and blue part of the spectrum (Figure S1).

## Conclusion

In conclusion, three π‐extended aza‐BODIPYs that absorb and emit in the far‐red and NIR regions of the electromagnetic spectrum have been synthesized. Two of them (aza‐FL, aza‐CZ) possess exceptional molar absorption coefficients and high quantum yields and thus belong to the brightest aza‐BODIPYs reported for this spectral region (Table S3). The highly favorable spectral properties and excellent photostability are strongly indicative of the potential for future biomedical applications. The potential of the new dyes for application in security inks was also demonstrated. The phenol receptor in aza‐OHCl induces pH sensitivity manifested as a drastic change in the absorption spectrum and complete fluorescence quenching upon deprotonation, thus suggesting its possible application as a new fluorescent/PA imaging probe. Further structural modifications of aza‐FL and aza‐CZ might be feasible (e. g., halogenation with Br or I, introduction of other receptor groups) to allow them to be adapted for use as triplet sensitizers for photodynamic therapy and organic upconversion or as PA probes.

## Experimental Section

**Materials**: Silica gel (0.04‐0.063 mm) was purchased from Acros Organics. 6‐Hydroxy‐1‐tetralone, triisopropylchlorosilane, 1*H*‐imidazole were from Fluorochem (www.fluorochem.co.uk). Lithium diisopropylamide (LDA; 2 M solution in THF), boron trifluoride etherate (BF_3_OEt_2_), *N*‐chlorosuccinimde, acetic anhydride, sodium nitrite and potassium carbonate, 5,5’‐dichloro‐11‐diphenylamino‐3,3’‐diethyl‐10,12‐ethylenethiatricarbocyanine perchlorate (IR‐140) and 1,1’,3,3,3’,3’‐hexamethylindotricarbocyanine iodide (HITC) were purchased from Sigma–Aldrich. *N,N*‐DIPEA and indocyanine green (IR‐125) were form TCI Europe (www.tcichemicals.com). Anhydrous sodium sulfate, hydrochloric acid (∼37 %), cyclohexane (CH), THF, and ethyl acetate (EA) were from VWR (www.vwr.com). Deuterated chloroform (CDCl_3_) and CD_2_Cl_2_ were from Eurisotrop (www.eurisotop.com). Propionic acid, the buffer salts (MES, HEPES), *N*,*N*‐dimethylformamide (DMF) were purchased from Roth. CHES was from Alfa Aesar. MOPS and dichloromethane were from Fischer Scientific. Eudragit®RL 100 copolymer poly(ethyl acrylate‐co‐methyl methacrylate‐co‐trimethylammonioethyl methacrylate chloride), 8.8‐12 % of quaternary ammonium groups, *M*_W_ ∼150 000 Da) was purchased from Degussa Germany (www.evonik.com).

**Measurements**: ^1^H NMR (300 MHz) and ^13^C NMR (76 MHz) spectra were recorded on a Bruker AVANCE III spectrometer (www.bruker.com). Note that the solubility of aza‐dipyrrins **2**, **4**, **10** and aza‐BOIDPYs **11**, aza‐FL, aza‐CZ and aza‐OHCl is too low to obtain ^13^C spectra of sufficient quality. Mass spectra were obtained on a matrix‐assisted laser desorption/ionization (MALDI)‐TOF/TOF (time of flight) spectrometer from Bruker. Absorption spectra were acquired on a Cary 50 UV/Vis spectrophotometer from Agilent Technologies. Emission spectra were recorded on a FluoroLog®3 spectrofluorometer from Horiba Scientific (www.horiba.com) equipped with a R2658 photomultiplier by Hamamatsu (300‐1050 nm; www.hamamatsu.com). Fluorescence lifetimes were measured with time correlated single photon counting (TCSPC) technique using DeltaHub module in combination with 453 or 635 nm laser diodes “NanoLED” (from Horiba) and DAS‐6 Analysis software for data analysis. Relative fluorescence quantum yields in THF were determined according to Demas and Crosby^[57]^ using ethanolic solutions of reference dyes IR‐125 (*ϕ*=0.132), IR‐140 (*ϕ*=0.167) and HITC (*ϕ*=0.283).^[45]^ The photostability of the dyes was accessed by irradiating stirred solutions of aza‐BODIPY dyes in THF or a solution of IR‐125 in ethanol (C=3.3x10^−6^ M) in a screw capped quartz cuvette (Hellma, www.hellma.com) with an array of 7 far‐red high‐power LEDs (*λ*=730 nm, OSRAM Oslon SSL 80, www.led‐tech.de) combined with a cooling block and a focusing lens from Edmund optics (www.edmundoptics.de). The photon flux of about ∼15 000 μmol s^−1^ m^−2^ was estimated with help of the Li‐250 A light meter equipped with Li‐190R quantum sensor (400‐700 nm) from Li–COR (www.licor.com). The measured photon flux for the part of the LED emission located in the visible range was ∼2000 μmol s^−1^ m^−2^; this value was corrected by the factor corresponding to the difference between the integrated emission of the LED in the range up to 700 nm and the total emission of the LED in the range from 650 to 775 nm. The absorption spectra were recorded every 10 min for a total of 90 min. The pH of the buffers (MOPS, MES, HEPPS, CHES) was adjusted with a pH meter (Mettler Toledo, Seven Easy pH/ion, www.mt.com) and the pH meter was calibrated with standard buffers of pH 4.01, pH 7.00 and pH 10.01 (DuraCal pH Buffer Solutions, www.hamiltoncompany.com). Fluorescent ink imaging was performed with a Jai AD 130 GE NIR/RGB Camera from Stemmer Imaging (www.stemmer‐imaging.com) by irradiating the sample with an array of 12 royal‐blue high‐power LEDs (*λ*=451 nm, OSRAM Oslon SSL 80).

**Synthesis**: 3‐Phenyl‐2*H*‐azirine **8**, 10‐isopropyl‐3‐phenyl‐4,10‐dihydro‐1*H*‐pyrrolo[3′,2′:4,5]cyclopenta[1,2‐b]carbazole **3** and 6,6‐dimethyl‐3‐phenyl‐4,6‐dihydro‐1*H*‐benzo[6,7]‐s‐indaceno[1,2‐b]pyrrole **1** were synthesized according to literature.[^44^, ^58^]

*Synthesis of aza‐BODIPY ligand (**2**)*: Sodium nitrite (0.187 g, 2.71 mmol, 20.0 equiv) was added at −10 °C to propionic acid (3 mL). After stirring of 10 min, a solution of **1** (0.047 g, 0.135 mmol, 1.0 equiv) in propionic acid (3 mL) was added. The suspension was stirred until completion of the reaction (as indicated by thin layer chromatography TLC). It was then diluted with water and extracted with CH_2_Cl_2_. The organic phase was washed three times with water and the solvent was removed under reduced pressure. The obtained brown nitrosyl pyrrole was again dissolved in propionic acid (3 mL) and was cooled to 0 °C. The second pyrrole moiety (0.047 g, 0.135 mmol, 1.0 equiv) in propionic acid (3 mL) was added, followed by addition of acetic anhydride (4.5 mL). After 1 h stirring, the reaction solution was extracted with dichloromethane and the organic phase was washed with water. The product was purified by column chromatography (silica gel) using CH+CH_2_Cl_2_ (2+1, *v*/*v*) as an eluent. The product was isolated as blue crystals (82.5 mg, 86 %). ^1^H NMR (300 MHz, CDCl_3_): *δ*=8.27 (s, 2H), 8.14 (d, J=7.2 Hz, 4H), 7.98 (d, 2H), 7.58 (s, 2H), 7.53–7.30 (m, 12H), 3.98 (s, 4H), 1.58 (s, 12H). MALDI‐TOF *m*/*z*: calcd. for: C_52_H_40_N_3_ [*M*H^+^]: 706.3222, found: 706.3245.

*Synthesis of aza‐BODIPY BF_2_ complex aza‐FL*: Compound **2** (75.6 mg, 0.107 mmol, 1.0 equiv) was dissolved in dry CH_2_Cl_2_, DIPEA (0.187 mL, 1.07 mmol, 10.0 equiv) was added under argon counter flow, and the reaction mixture was stirred for 15 min. After addition of BF_3_OEt_2_ (0.204 mL, 1.61 mmol, 15.0 equiv) under argon counter flow, the mixture was again stirred for 15 min and was extracted with H_2_O and CH_2_Cl_2_, the organic phase was dried over Na_2_SO_4_ and the solvent was removed. The product was precipitated using CH_2_Cl_2_/EA as coppery crystals. (58 mg, 72 %). ^1^H NMR (300 MHz, CDCl_3_): *δ*=8.69 (s, 2H), 8.18 (d, *J*=7.1 Hz, 4H), 8.10 (d, 2H), 7.63 (s, 2H), 7.57–7.38 (m, 12H), 4.05 (s, 4H), 1.59 (s, 12H). MALDI‐TOF *m*/*z*: calcd. for: C_52_H_38_BF_2_N_3_ [*M* ^+^]: 753.3136, found: 753.3060.

*Synthesis of aza‐BODIPY ligand (**4**)*: The synthesis of **4** was performed analogously to that of aza‐BODIPY ligand **2** but 46 mg (0.126 mmol, 1.0 equiv) of **3** were used instead. The product was isolated as green crystals (35 mg, 75 %). ^1^H NMR (300 MHz, CD_2_Cl_2_): *δ*=8.24 (s, 2H), 8.15 (q, *J*=7.9, 6.6 Hz, 8H), 7.59 (d, *J*=7.6 Hz, 2H), 7.50 (t, *J*=7.2 Hz, 6H), 7.38 (t, *J*=7.3 Hz, 2H), 7.25 (t, *J*=7.4 Hz, 2H), 5.25–5.07 (m, 2H), 4.13 (s, 4H), 1.86 (d, *J*=4.1 Hz, 12H). MALDI‐TOF *m*/*z*: calcd. for: C_52_H_42_N_5_ [*M*H^+^]: 736.3440, found: 736.3411.

*Synthesis of aza‐BODIPY complex aza‐CZ*: The synthesis of aza‐CZ was performed analogously to that of aza‐FL but 27.5 mg (0.037 mmol, 1.0 equiv) of aza‐BODIPY ligand **4** were used instead. The product was purified by column chromatography on silica gel using CH+CH_2_Cl_2_ (2+1, *v*/*v*) as an eluent and was isolated as black crystals (25 mg, 86 %). ^1^H NMR (300 MHz, CD_2_Cl_2_): *δ*=8.61 (s, 2H), 8.28–8.20 (m, 6H), 8.14 (d, *J*=7.5 Hz, 2H), 7.59–7.48 (m, 8H), 7.48–7.38 (m, 2H), 7.24 (t, *J*=7.2 Hz, 2H), 5.16 (dt, *J*=13.1, 6.6 Hz, 2H), 4.15 (s, 4H), 1.90 (d, *J*=6.9 Hz, 12H). MALDI‐TOF *m*/*z*: calcd. for: C_52_H_40_BF_2_N_5_ [*M* ^+^]:783.3354, found: 783.3966.

*Synthesis of 5‐chloro‐6‐hydroxy‐3,4‐dihydronaphthalen‐1(2H)‐one (**6**)*: 6‐Hydroxy‐1‐tetralone **5** (2.00 g, 12.33 mmol, 1.0 equiv) was dissolved in DMF (60 mL), and a solution of *N*‐chlorosuccinimide (3.29 g, 24.66 mmol, 2.0 equiv) in DMF (60 mL) was added dropwise. The mixture was stirred at 70 °C for 3 h and was extracted with H_2_O and CH_2_Cl_2_, the organic phase was dried over Na_2_SO_4_, and the solvent was removed. The product was purified by column chromatography (silica gel) using CH+EA (8+1, *v*/*v*) as an eluent. The product was isolated as a beige powder (1.47 g, 61 %).^1^H NMR (300 MHz, CDCl_3_): *δ*=7.97 (d, *J*=8.6 Hz, 1H), 7.00 (d, *J*=8.6 Hz, 1H), 6.18 (s, 1H), 3.00 (t, *J*=6.1 Hz, 2H), 2.61 (t, 2H), 2.15 (q, 2H).^13^C NMR (76 MHz, CDCl_3_): *δ*=196.42, 155.65, 143.29, 127.91, 127.29, 118.90, 114.41, 37.96, 27.40, 22.40.

*Synthesis of 5‐chloro‐6‐((triisopropylsilyl)oxy)‐3,4‐dihydronaphthalen‐1(2H)‐one (**7**)*: 1*H*‐Imidazole (0.258 g, 3.79 mmol, 1.1 equiv) was dissolved in dry THF (60 mL) and triisopropylchlorosilane (0.812 mL, 3.79 mmol, 1.1 equiv) was added dropwise and was stirred for about 20 min. After the addition of **6** (0.678 g, 3.45 mmol, 1.0 equiv), the mixture was stirred overnight and extracted with H_2_O and CH_2_Cl_2_, the organic phase was dried over Na_2_SO_4_, and the solvent was removed. The product was purified by column chromatography (silica gel) using CH+EA (10+1, *v*/*v*) as an eluent. The product was isolated as yellow oil (0.58 g, 50 %).^1^H NMR (300 MHz, CD_2_Cl_2_): *δ*=7.85 (d, *J*=8.6 Hz, 1H), 6.88 (d, *J*=8.6 Hz, 1H), 3.01 (t, *J*=6.1 Hz, 2H), 2.56 (t, 2H), 2.13 (q, *J*=6.4 Hz, 2H), 1.40 ‐ 1.28 (m, 3H), 1.13 (d, *J*=7.3 Hz, 18H). ^13^C NMR (76 MHz, CD_2_Cl_2_): *δ*=196.62, 156.73, 144.61, 127.92, 127.06, 124.65, 118.09; 38.45, 28.02, 18.09, 13.39.

*Synthesis of 6‐chloro‐3‐phenyl‐7‐((triisopropylsilyl)oxy)‐4,5‐dihydro‐1H‐benzo[g]indole (**9**)*: **7** (0.580 g, 1.64 mmol, 1.0 equiv) was dissolved in dry THF (12 mL) and cooled down to −78 °C. LDA (2 M in THF; 0.90 mL, 1.80 mmol, 1.1 equiv) and 3‐phenyl‐2*H*‐azirene **8** (0.21 mg, 1.80 mmol, 1.1 equiv) were added dropwise under Ar counterflow. The mixture was stirred for 3 h at −78 °C, warmed up to RT, quenched with water and neutralized with diluted HCl (0.05 M). THF was removed and the mixture was extracted with CH_2_Cl_2_. The organic phase was dried over Na_2_SO_4_, the solvent was removed, and the product was purified by column chromatography (silica gel) using CH+EA (10+1, *v*/*v*) as an eluent. The product was isolated as a yellow powder (0.35 g, 47 %). ^1^H NMR (300 MHz, CD_2_Cl_2_): *δ*=8.43 (s, 1H), 7.45 (d, *J*=7.1 Hz, 2H), 7.37 (t, 2H), 7.22 (t, *J*=7.2 Hz, 1H), 7.02 (d, *J*=8.3 Hz, 1H), 6.97 (d, *J*=2.5 Hz, 1H), 6.82 (d, *J*=8.3 Hz, 1H), 3.11 (t, *J*=7.5 Hz, 2H), 2.93 (t, *J*=7.4 Hz, 2H), 1.42 ‐ 1.26 (m, 3H), 1.15 (d, *J*=7.2 Hz, 18H). ^13^C NMR (76 MHz, CD_2_Cl_2_): *δ*=150.35, 136.33, 134.58, 128.93, 128.55, 127.35, 125.97, 124.20, 123.99, 117.67, 117.44, 116.58, 116.35, 107.56, 27.60, 21.11, 18.17, 13.39.

*Synthesis of aza‐BODIPY ligand (**10**)*: The synthesis of **10** was performed analogously to that of aza‐BODIPY ligand **2** but 35 mg (0.10 mmol, 1.0 equiv) of pyrrole **3** were used instead of pyrrole **1**. The resulted nitroso‐derivate was reacted with 44 mg (0.10 mmol, 1.0 equiv) of pyrrole **9**. The product was isolated as golden crystals (50 mg, 63 %). ^1^H NMR (300 MHz, CD_2_Cl_2_): *δ*=8.23 (s, 1H), 8.15 (d, *J*=8.7 Hz, 3H), 8.07 (s, 1H), 7.82–7.73 (m, 3H), 7.63 (d, *J*=7.5 Hz, 1H), 7.55–7.45 (m, 3H), 7.45–7.18 (m, 5H), 7.06 (d, *J*=7.5 Hz, 1H), 5.23–5.13 (m, 1H), 4.15 (s, 2H), 1.82 (d, *J*=7.0 Hz, 6H), 1.33–1.25 (m, 3H), 1.20 (d, *J*=7.3 Hz, 18H). MALDI‐TOF *m*/*z*: calcd. for: C_52_H_39_N_3_ [*M*H^+^]: 825.3755, found: 825.3781.

*Synthesis of aza‐BODIPY complex (**11**)*: The synthesis of **11** was performed analogously to that of aza‐FL but 22.5 mg (0.027 mmol, 1.0 equiv) of aza‐BODIPY ligand **10** were used instead. The product was isolated as black crystals (17 mg, 71 %). ^1^H NMR (300 MHz, CDCl_3_): *δ*=8.72 (d, *J*=8.9 Hz, 1H), 8.48 (s, 1H), 8.18 (d, *J*=7.1 Hz, 2H), 8.11 (s, 1H), 8.06 (d, *J*=7.7 Hz, 1H), 7.75 (d, *J*=7.0 Hz, 2H), 7.44 (ddt, *J*=29.0, 14.6, 7.0 Hz, 8H), 7.20 (t, *J*=6.7 Hz, 1H), 7.06 (d, *J*=8.8 Hz, 1H), 5.09 (dt, *J*=13.7, 6.6 Hz, 1H), 4.07 (s, 2H), 3.16 (t, *J*=6.6 Hz, 2H), 2.95 (t, *J*=6.7 Hz, 2H), 1.80 (d, *J*=6.9 Hz, 7H), 1.42 (dq, *J*=13.7, 7.2, 6.7 Hz, 3H), 1.22 (d, *J*=7.2 Hz, 18H). MALDI‐TOF *m*/*z*: calcd. for: C_53_H_52_BClF_2_N_4_OSi [*M*H^+^]: 873.3748, found: 873.3679.

*Synthesis of aza‐BODIPY complex aza‐OHCl*: Compound **11** (15.5 mg, 0.018 mmol, 1.0 equiv) was dissolved in DMF (6 mL), and KOAc (87.1 mg, 0.887 mmol, 50.0 equiv) was added. The reaction solution was stirred for 1 h and was extracted with water and CH_2_Cl_2_, the organic phase was dried over Na_2_SO_4_ and the solvent was removed. The product was obtained as black crystals (10 mg, 79 %). ^1^H NMR (300 MHz, CD_2_Cl_2_): *δ*=8.76 (d, *J*=9.0 Hz, 1H), 8.48 (s, 1H), 8.24 (d, *J*=7.7 Hz, 3H), 8.15 (d, *J*=7.5 Hz, 1H), 7.78 (d, *J*=7.9 Hz, 2H), 7.62–7.39 (m, 8H), 7.31–7.15 (m, 2H), 6.14 (s, 1H), 5.23–5.02 (m, 1H), 4.18 (s, 2H), 3.16 (t, *J*=7.7 Hz, 2H), 2.98 (t, 2H), 1.82 (d, *J*=6.9 Hz, 6H). MALDI‐TOF *m*/*z*: calcd. for: C_44_H_32_BClF_2_N_4_O [*M*H^+^]: 717.2411, found: 717.2501.

*Preparation of the RL‐100 particles*: 30 mg of Eudragit RL‐100 (0.2 % *w*/*w* solution) and aza‐CZ (0.75 % *w*/*w* in respect to the polymer) were dissolved in 15 g of a 1 : 1 *w*/*w* solvent mixture of acetone and THF. 50 mL water was added quickly under vigorous stirring (∼10 mL s^−1^) and the organic solvents were removed under reduced pressure and was further concentrated to a volume of 40 mL. For application as security ink the dispersion was diluted with water to the final concentration of 0.15 mg/mL.

## Conflict of interest

The authors declare no conflict of interest.

## Supporting information

As a service to our authors and readers, this journal provides supporting information supplied by the authors. Such materials are peer reviewed and may be re‐organized for online delivery, but are not copy‐edited or typeset. Technical support issues arising from supporting information (other than missing files) should be addressed to the authors.

Supporting Information
