# Supplementary material for: Carbazole‐ and Fluorene‐Fused Aza‐BODIPYs: NIR Fluorophores with High Brightness and Photostability
Source: Chemistry. 2021 Jun 2;27(41):10685–92. doi: 10.1002/chem.202100965 (PMC8362076; doi:10.1002/chem.202100965)
Supplement: Supplementary file 1 — Supporting Information [file CHEM-27-10685-s001.pdf]

Chemistry–A European Journal

Supporting Information

Carbazole- and Fluorene-Fused Aza-BODIPYs: NIR Fluorophores with High Brightness and Photostability

Tanja Rappitsch and Sergey M. Borisov*

Contents

Photophysical Properties 2

Photobleaching 3

Table S 1. Photophysical Properties of new aza-BODIPYs and **TPAB** in different solvents at room temperature. 5

Table S 2. Examples of NIR emitting dyes 5

Table S 3. Summary of photophysical properties of reported aza-BODIPY dyes 7

NMR Data 15

MS Data 22

References 26

Photophysical Properties

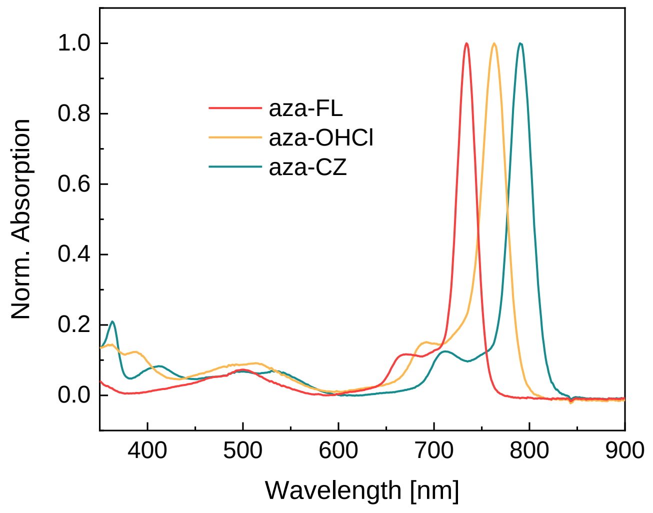

Figure S 1. Absorption spectra of **aza-FL, aza-OHCl and aza-CZ** from 350-900 nm in THF.

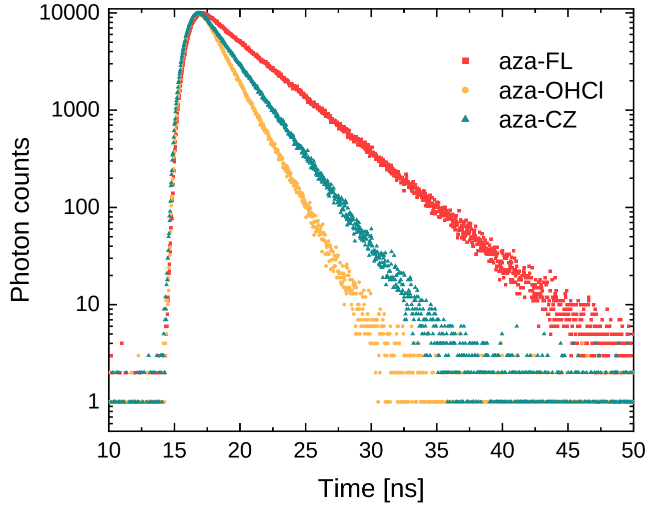

Figure S 2. Fluorescence decays of **aza-FL**, **aza-OHCl** and **aza-CZ** in THF.

Photobleaching

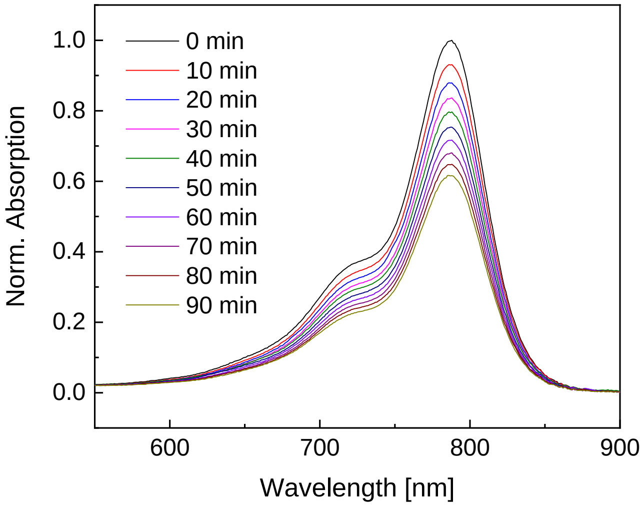

Figure S 3. Absorption spectra of **IR-125** in ethanol upon irradiation with a high-power LED array (λexc 730 nm).

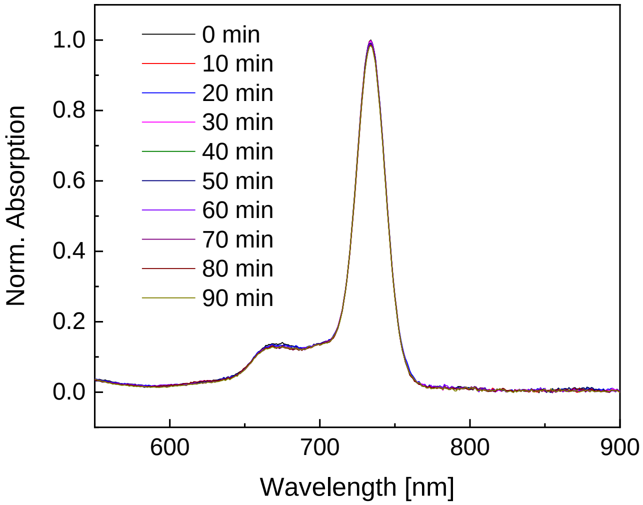

Figure S 4. Absorption spectra of **aza-FL** in THF upon irradiation with a high-power LED array (λexc 730 nm).

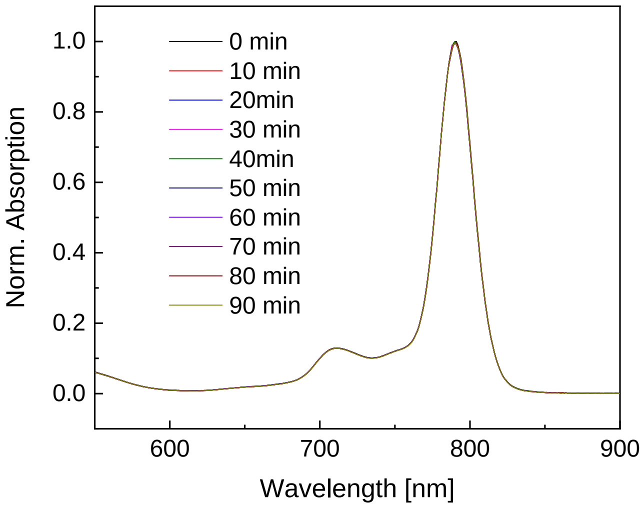

Figure S 5. Absorption spectra of **aza-CZ** in THF upon irradiation with a high-power LED array (λexc 730 nm).

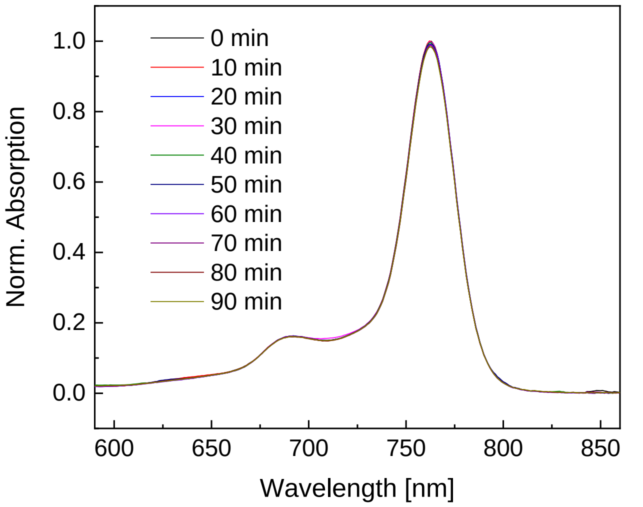

Figure S 6. Absorption spectra of **aza-OHCl** in THF upon irradiation with a high-power LED array (λexc 730 nm).

Table S 1. Photophysical Properties of new aza-BODIPYs and **TPAB** in different solvents at room temperature.

Dye	Solvent	λ_{abs} [nm]	λ_{em} [nm]	ϕ_F	Lifetime [ns]
**aza-FL**	THF	734	741	0.66	4.3
	toluene	741	748	0.65	4.5
	acetonitrile	731	739	0.67	4.8
**aza-CZ**	THF	791	800	0.49	2.3
	toluene	795	802	0.59	2.8
	acetonitrile	786	799	0.23	1.3
**aza-OHCl**	THF	763	776	0.16	1.7
	toluene	763	773	0.17	1.9
	acetonitrile	757	770	0.04	0.6
**TPAB**	$CHCl_3$	650	674	0.34	0.9
	toluene	654	678	0.39	1.8
	acetonitrile	644	668	0.14	0.6

Table S 2. Examples of NIR emitting dyes

maxima of absorption and emission (λabs and λem, respectively), molar absorption coefficients ε and fluorescence quantum yields ϕ.

Dye structure	λ_{abs} [nm]	λ_{em} [nm]	ε [M^{-1} cm^{-1}]	ϕ	Solvent	Reference
**Cyanines**						
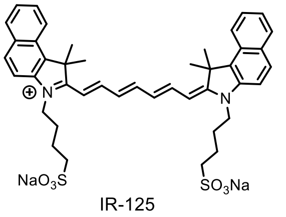 IR-125	787	818	194 171	0.132	EtOH	[1]
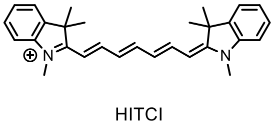 HITCI	743	773	251 929	0.283	EtOH	[1]

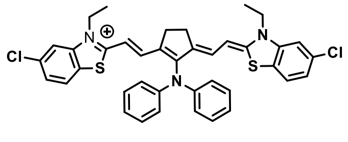<br>IR-140	804	844	173 919	0.167	EtOH	[1]
**Pyrrolopyrrole Cyanines**						
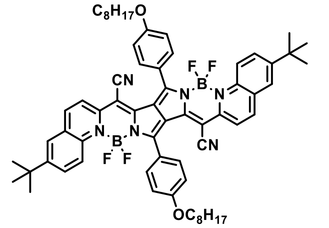	754	773	205 000	0.59	$CHCl_3$	[2]
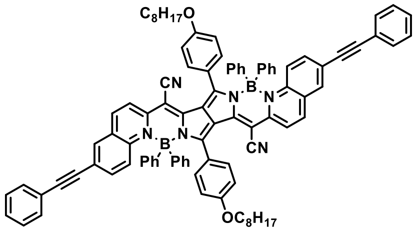	843	855	309 000	0.36	$CHCl_3$	[3]
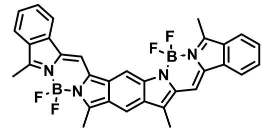	848	868	204 000	0.04	$CHCl_3$	[4]
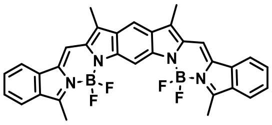	775	781	141 000	0.36	$CHCl_3$	[4]
**Squaraines**						
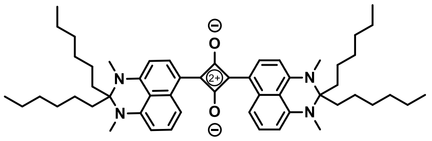	737	751	200 000	0.56	cyclohexane	[5]
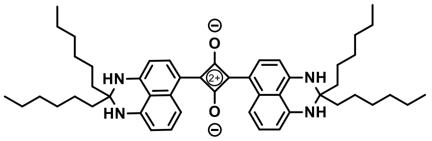	802	811	140 000	0.067	cyclohexane	[5]
**Modified Rhodamines**						
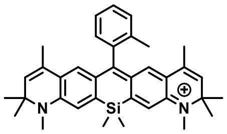	721	740	160 000	0.05	PBS, pH 7.4	[6]
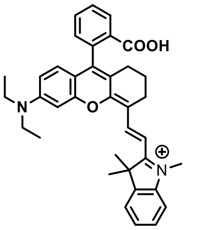	700	731	139 500	0.41	EtOH	[7]

Table S 3. Summary of photophysical properties of reported aza-BODIPY dyes

**Dye structure**	**λ_{abs} [nm]**	**λ_{em} [nm]**	**ε [M^{-1} cm^{-1}]**	**ϕ**	**Solvent**	**Reference**
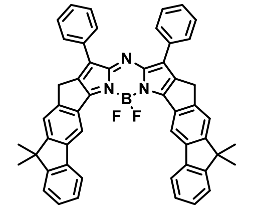	734	741	228 600	0.66	THF	This work
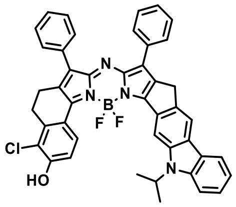	763	776	161 200	0.16	THF	This work
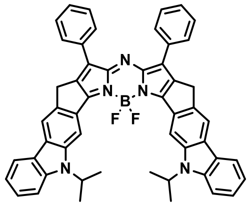	791	800	236 600	0.49	THF	This work
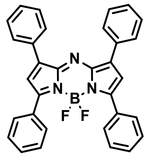	650	672	79 000	0.34	$CHCl_3$	[8]
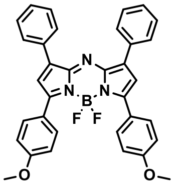	688	715	85 000	0.36	$CHCl_3$	[9]
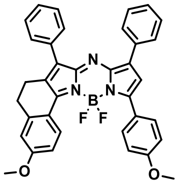	708	732	96 200	0.38	$CHCl_3$	[10]
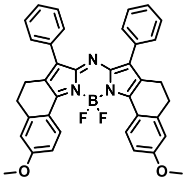	740	752	159 000	0.28	$CHCl_3$	[10]
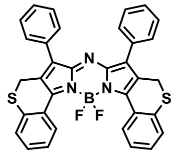	706	730	115 000	0.11	$CHCl_3$	[10]

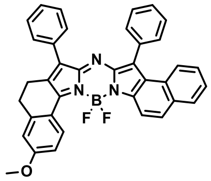	715	730	141 000	0.11	$CHCl_3$	[10]
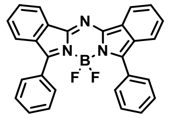	712	736	95 000	0.14	CH_2Cl_2	[11]
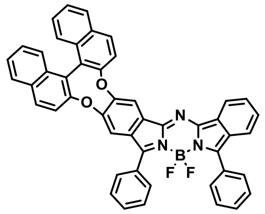	717	749	100 000	0.11	CH_2Cl_2	[12]
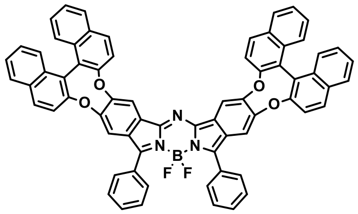	722	754	109 600	0.08	CH_2Cl_2	[12]
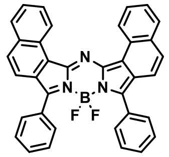	737	753	71 000	0.14	CH_2Cl_2	[11]
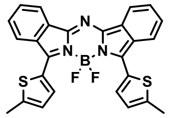	793	841	95 000	N.A.	CH_2Cl_2	[13]
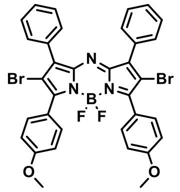	679	714	75 000	0.10	$CHCl_3$	[9]
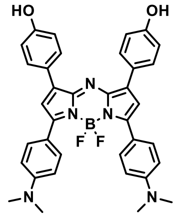	778	813	N.A.	0.10	toluene	[14]
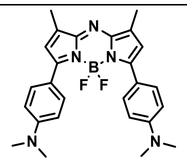	734	779	N.A.	0.21	toluene	[14]

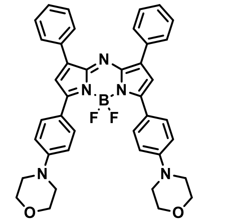	764	828	86 000	0.02	CH_2Cl_2	[15]
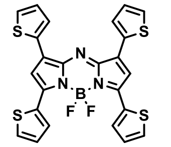	733	757	103 100	0.11	acetonitrile	[16]
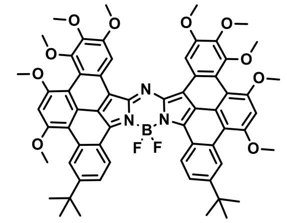	878	907	260 100	0.13	toluene	[17]
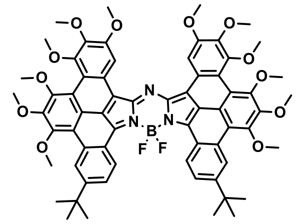	850	873	235 400	<0.01	toluene	[17]
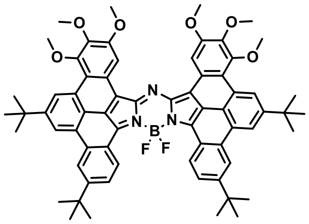	826	832	449 400	0.07	toluene	[17]
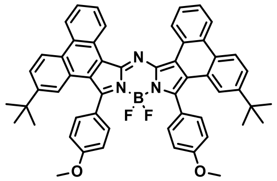	716	741	147 700	0.32	toluene	[18]
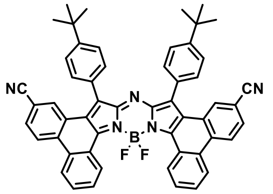	747	762	132 700	0.12	toluene	[19]
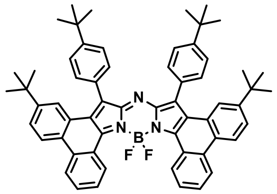	771	807	75 500	0.01	toluene	[19]

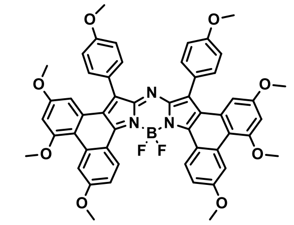	804	816	215 900	0.13	toluene	[20]
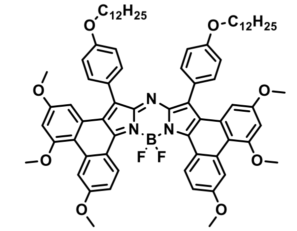	804	816	235 100	0.17	toluene	[20]
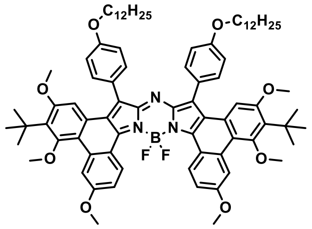	790	807	208 100	0.05	toluene	[20]
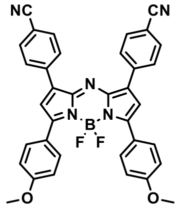	720	754	N.A.	0.36	toluene	[21]
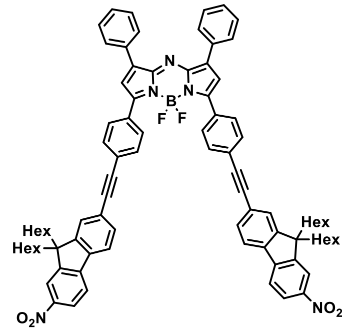	694	741	100 000	0.36	CH_2Cl_2	[22]
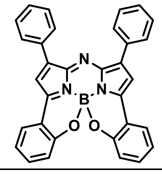	728	746	N.A.	0.51	toluene	[23]
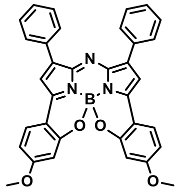	765	782	N.A.	0.18	$CHCl_3$	[23]

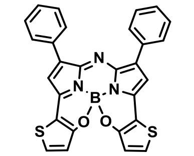	780	808	56 100	0.04	CH_2Cl_2	[24]
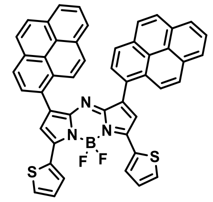	738	766	74 130	0.03	toluene	[25]
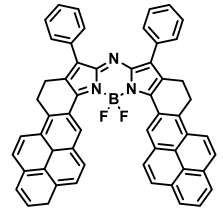	746	762	148 000	0.16	CH_2Cl_2	[26]
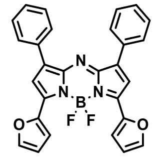	721	730	133 900	0.22	CH_2Cl_2	[27]
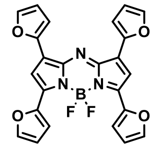	751	758	130 700	0.08	CH_2Cl_2	[27]
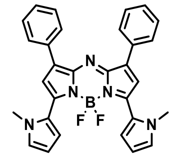	754	803	110 000	0.26	CH_2Cl_2	[28]
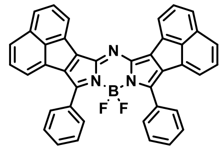	674	755	41 300	0.01	toluene	[29]
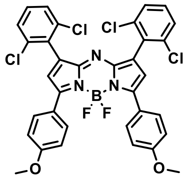	691	723	95 500	0.67	toluene	[30]
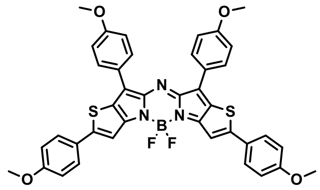	788	814	170 000	0.10	$CHCl_3$	[31]

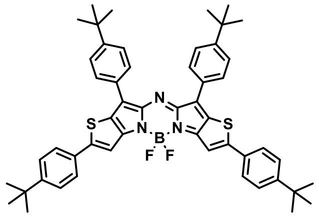	767	793	223 900	0.12	$CHCl_3$	[31]
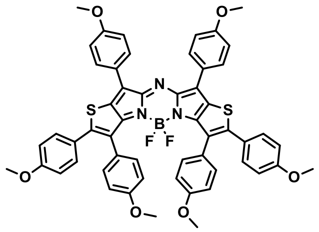	784	816	182 000	0.04	$CHCl_3$	[31]
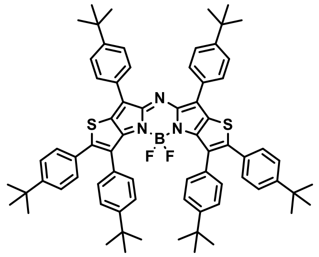	767	807	144 500	0.05	$CHCl_3$	[31]
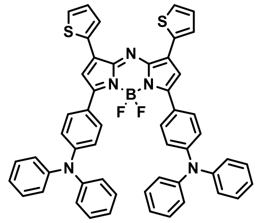	818	896	15 200	0.14	$CHCl_3$	[32]
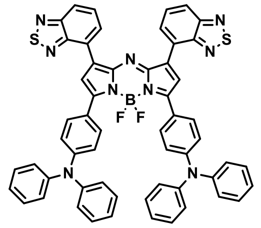	855	953	25 400	0.01	$CHCl_3$	[32]
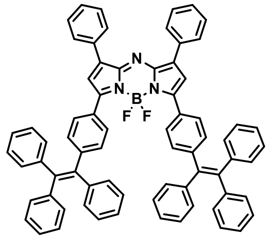	708	754	77 500	0.45	toluene	[33]
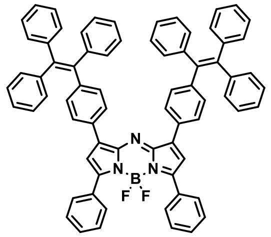	682	730	77 100	0.15	toluene	[33]

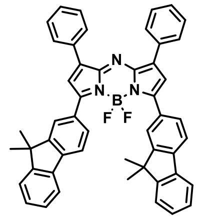	708	742	92 400	0.42	toluene	[33]
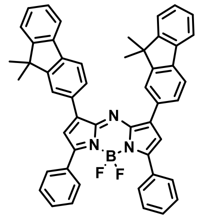	677	713	86 700	0.19	toluene	[33]
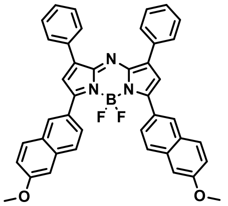	706	733	89 000	0.33	$CHCl_3$	[34]
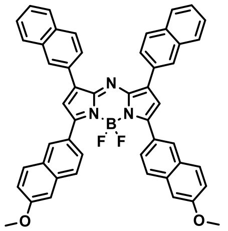	718	754	95 000	0.29	CH_2Cl_2	[35]
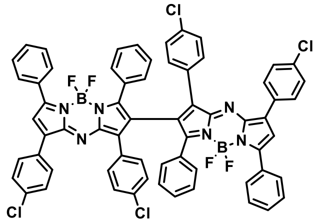	702	736	75 900	0.10	THF	[36]
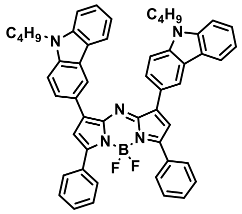	674	711	67 600	0.31	toluene	[37]
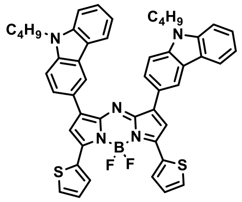	735	744	138 000	0.09	toluene	[37]

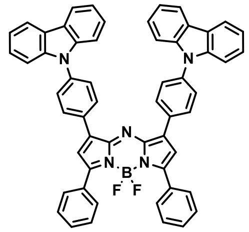	712	756	43 700	0.04	toluene	[37]
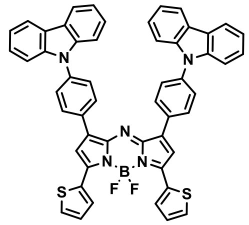	750	771	87 100	0.04	toluene	[37]
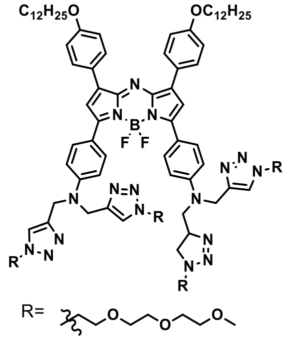	781	836	130 000	0.29	CH_2Cl_2	[38]
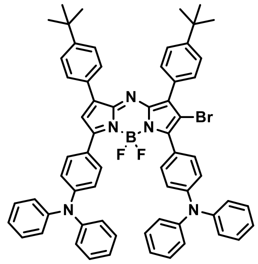	769	838	83 000	0.006	THF	[39]
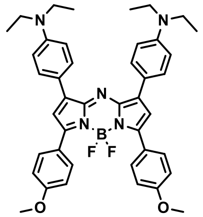	776	907	75 000	N.A	THF	[40]
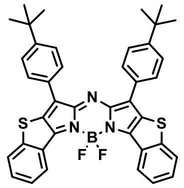	709	800	67 100	< 0.01	THF	[41]
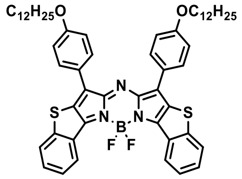	729	813	79 900	< 0.01	THF	[41]

NMR Data

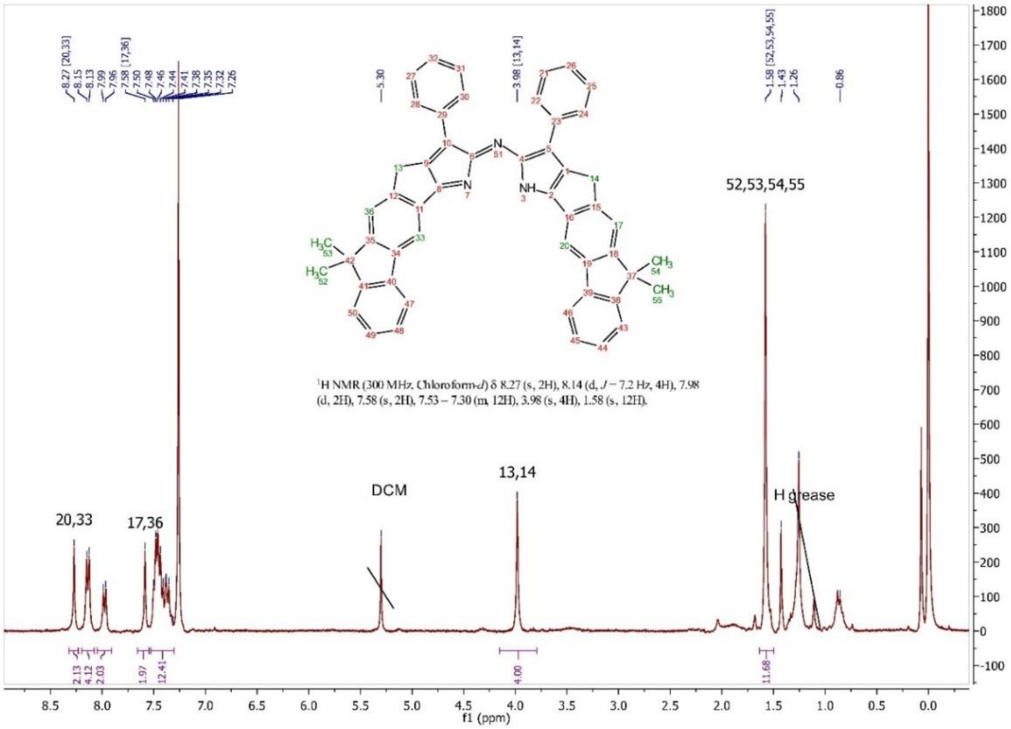

Figure S 7. ^{1}H NMR (300 MHz, $CDCl_3$) of **2**.

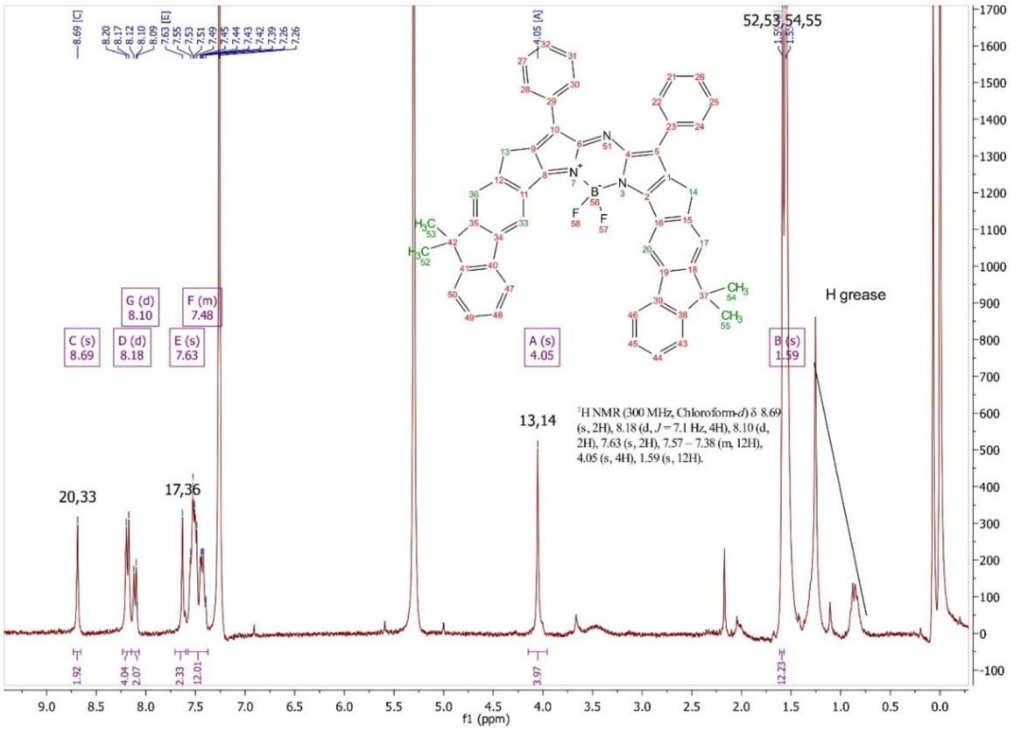

Figure S 8. ^{1}H NMR (300 MHz, $CDCl_3$) of **aza-FL**.

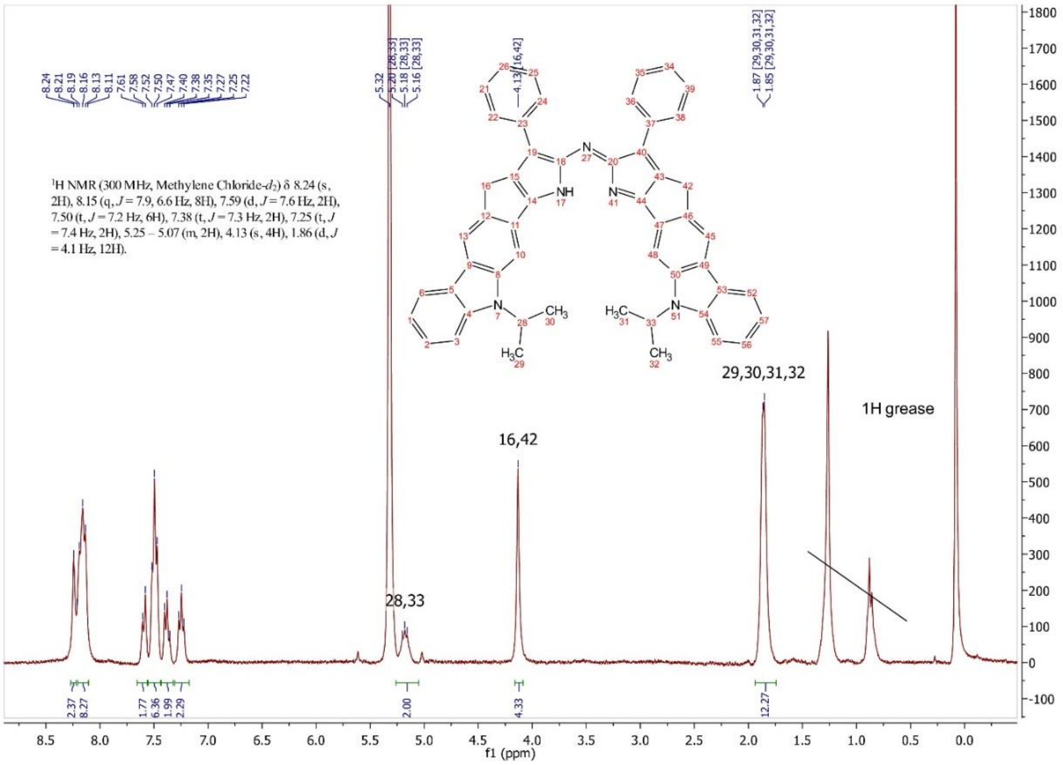

Figure S 9. ^{1}H NMR (300 MHz, CD_2Cl_2) of **4.**

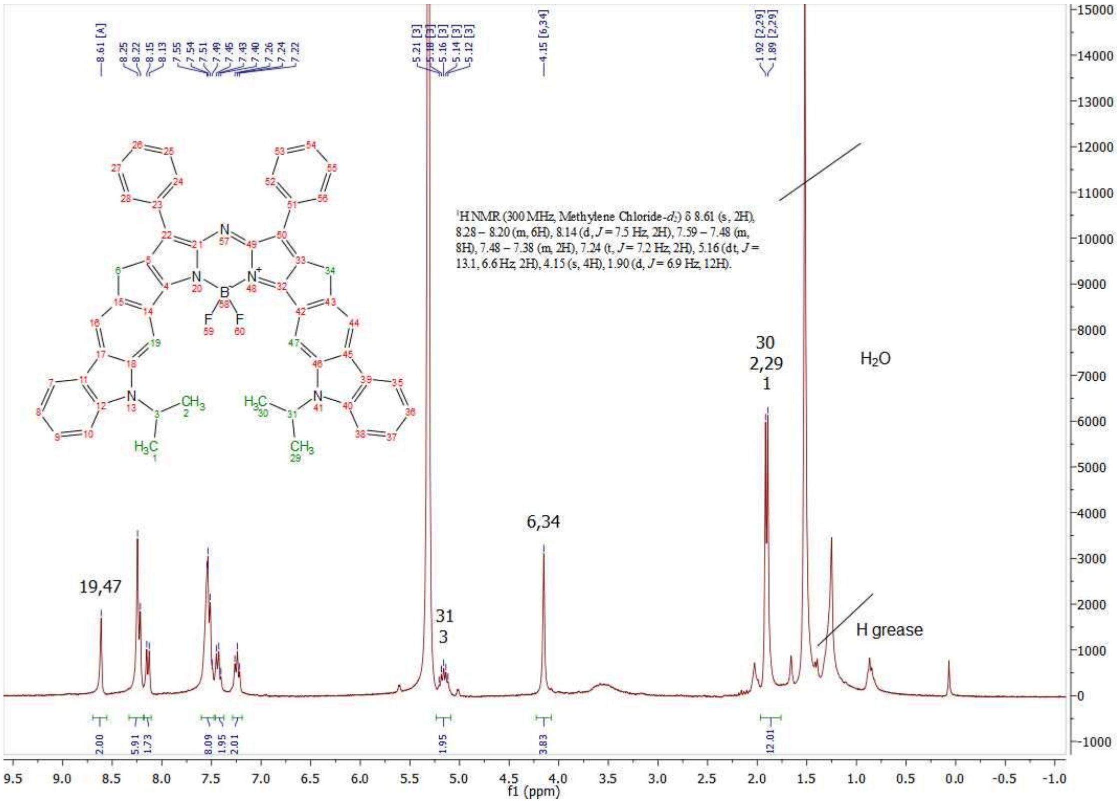

Figure S 10. ^{1}H NMR (300 MHz, CD_2Cl_2) of **aza-CZ.**

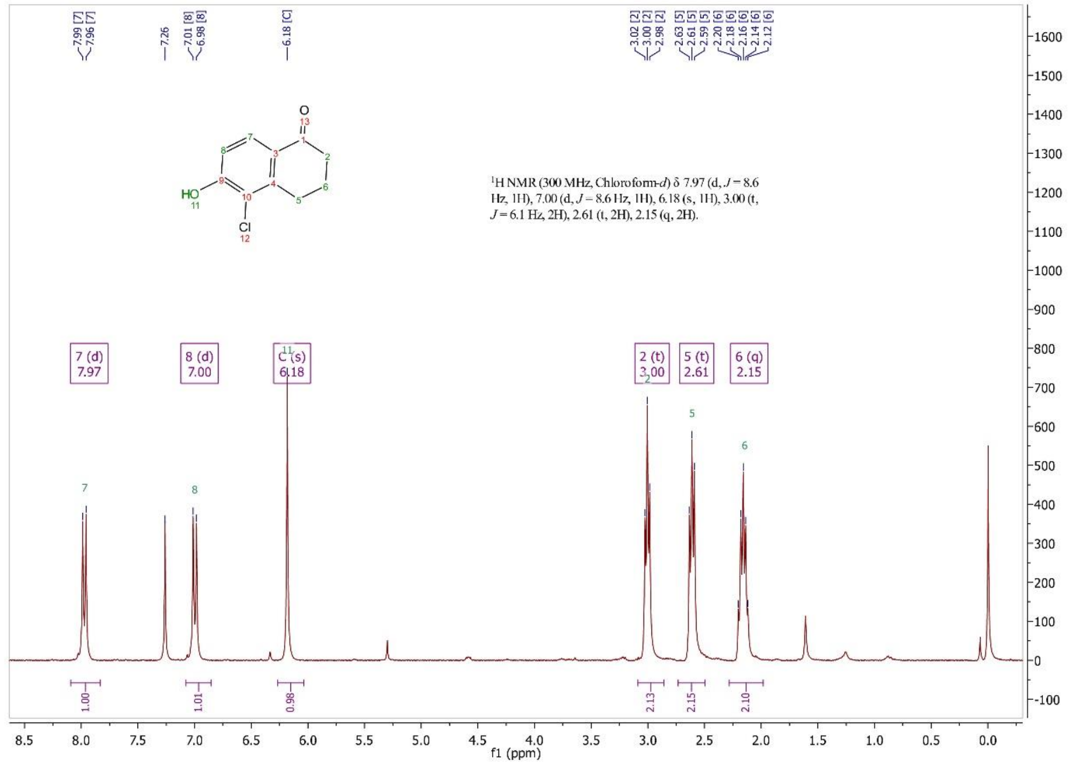

Figure S 11. ^{1}H NMR (300 MHz, $CDCl_3$) of **6.**

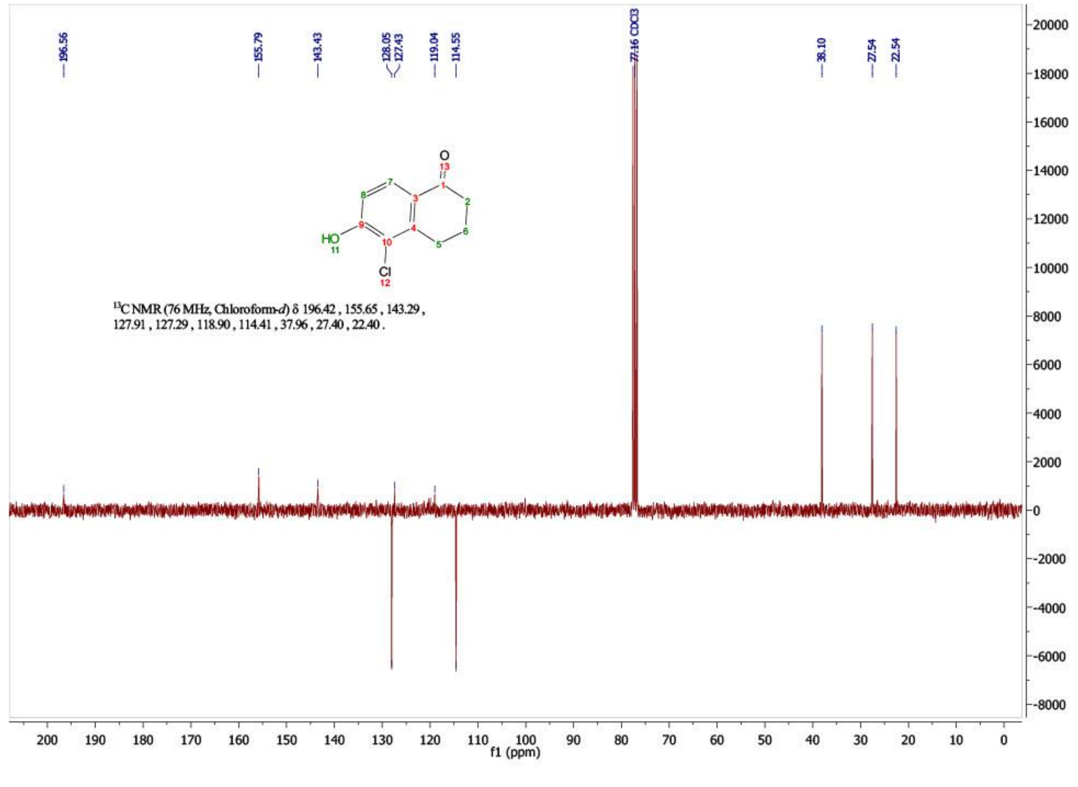

Figure S 12. ^{13}C-APT NMR (76 MHz, $CDCl_3$) of **6.**

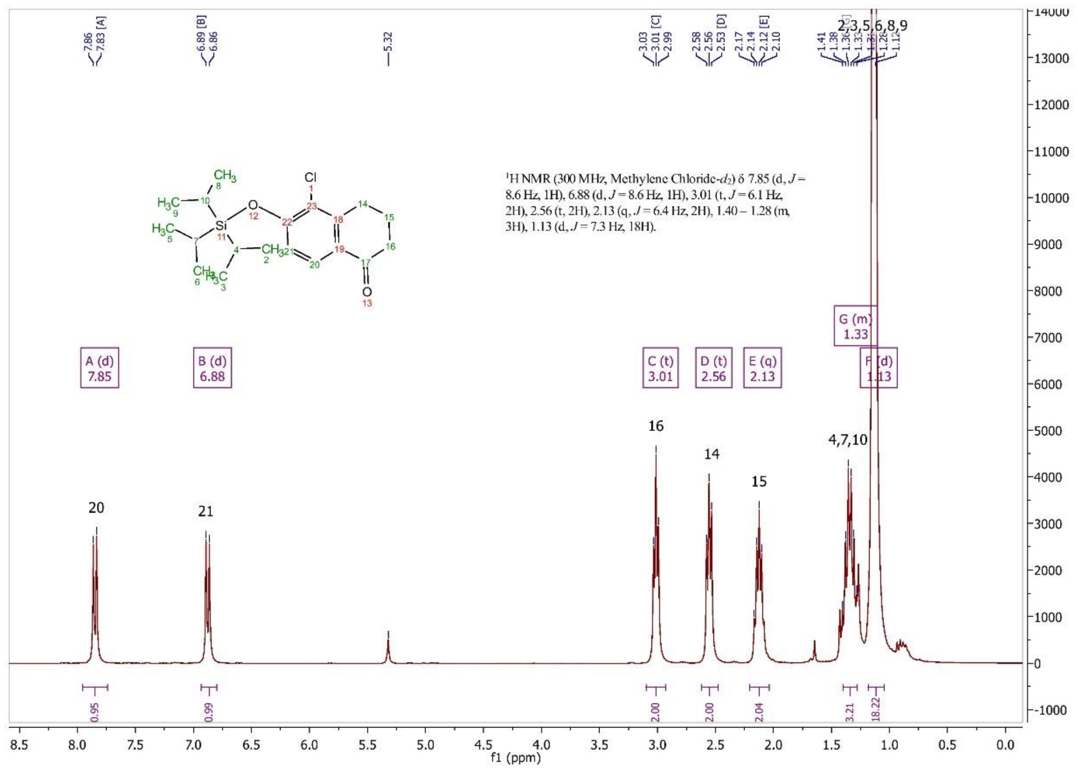

Figure S 13. ^{1}H NMR (300 MHz, CD_2Cl_2) of **7.**

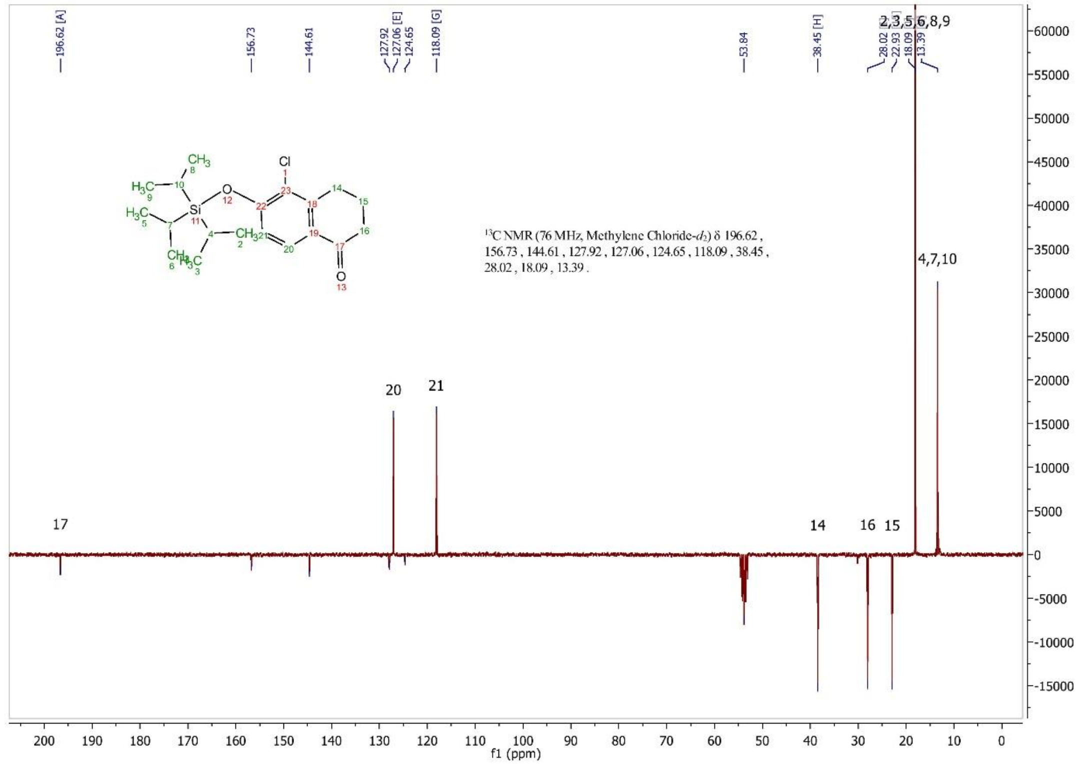

Figure S 14. ^{13}C-APT NMR (300 MHz, CD_2Cl_2) of **7.**

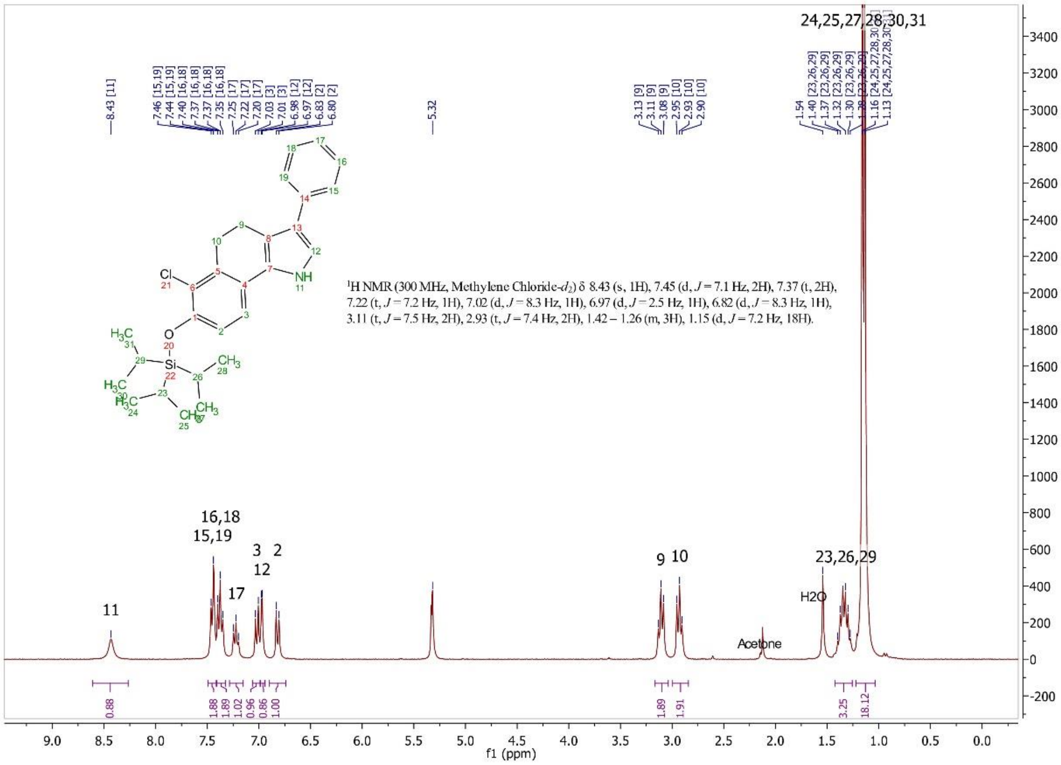

Figure S 15. ^{1}H NMR (300 MHz, CD_2Cl_2) of **9.**

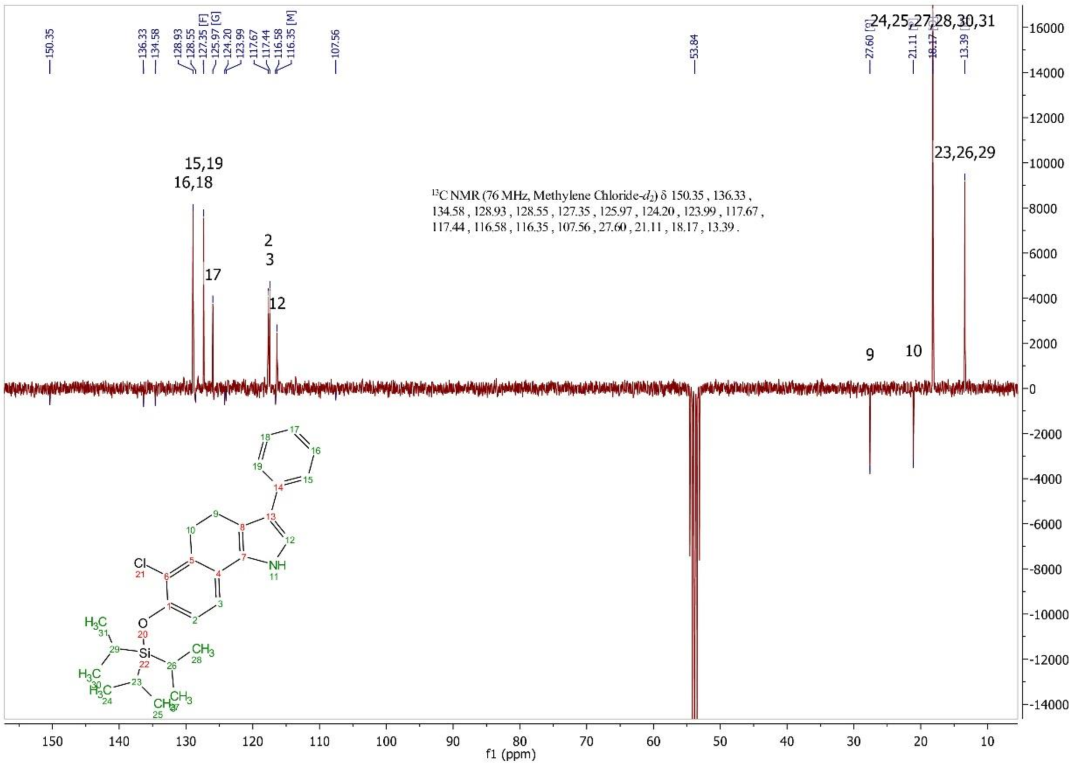

Figure S 16. ^{13}C-APT NMR (300 MHz, CD_2Cl_2) of **9.**

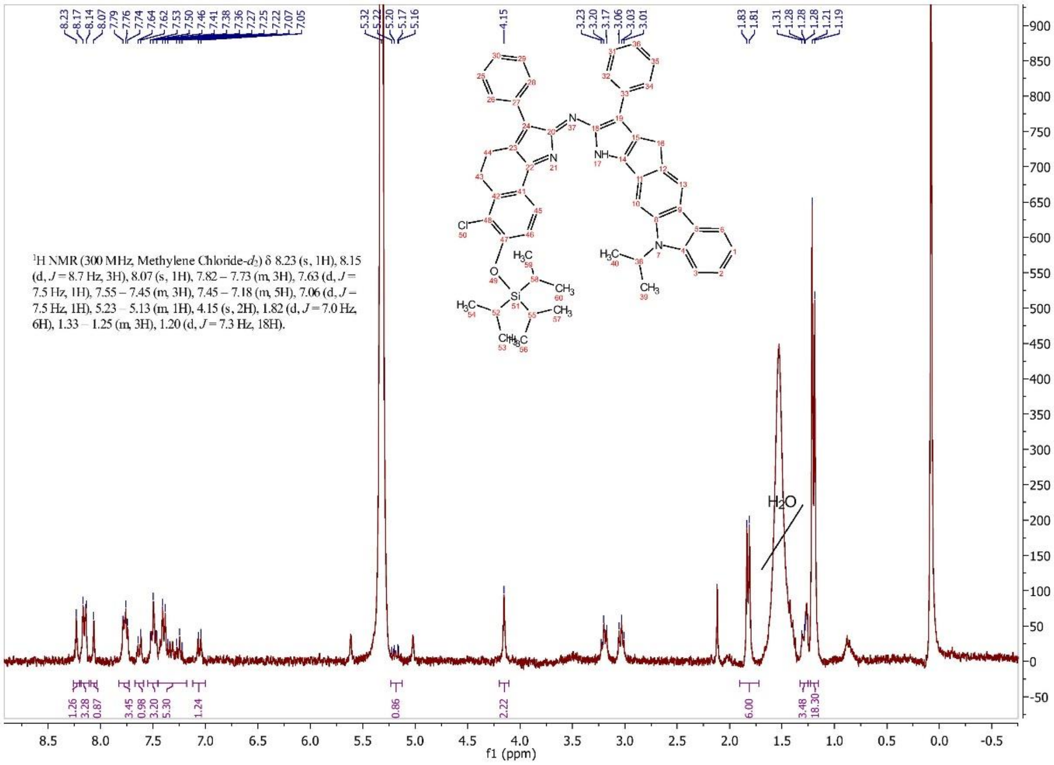

Figure S 17. ^{1}H NMR (300 MHz, CD_2Cl_2) of **10.**

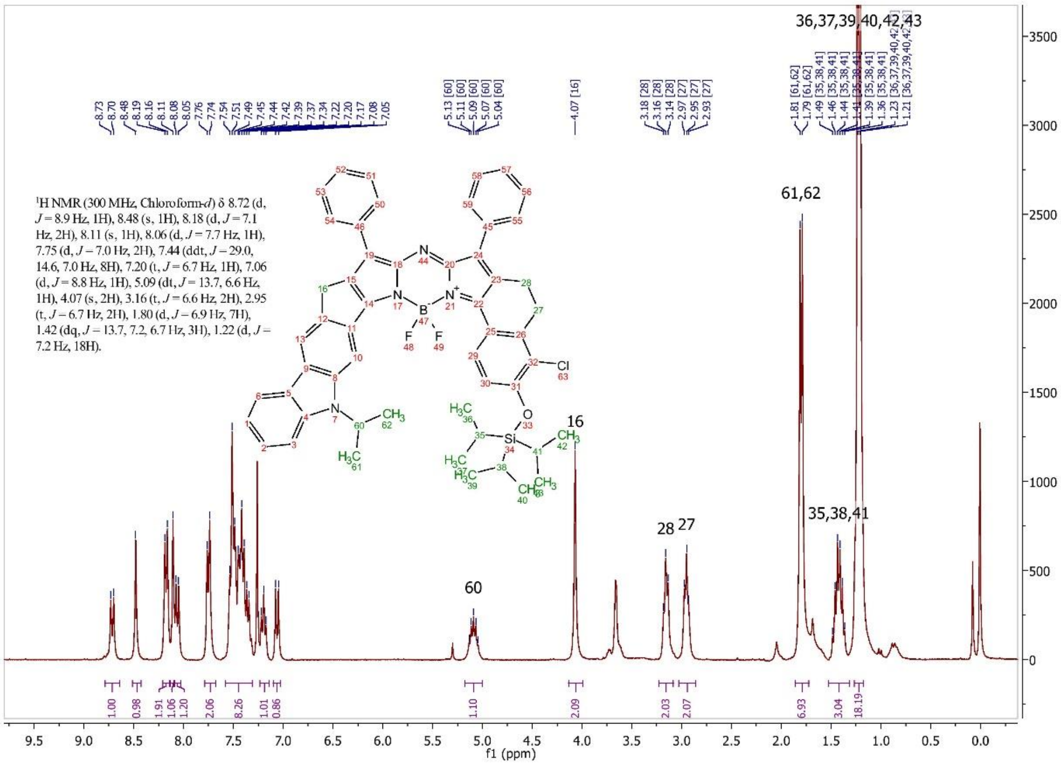

Figure S 18. ^{1}H NMR (300 MHz, $CDCl_3$) of **11.**

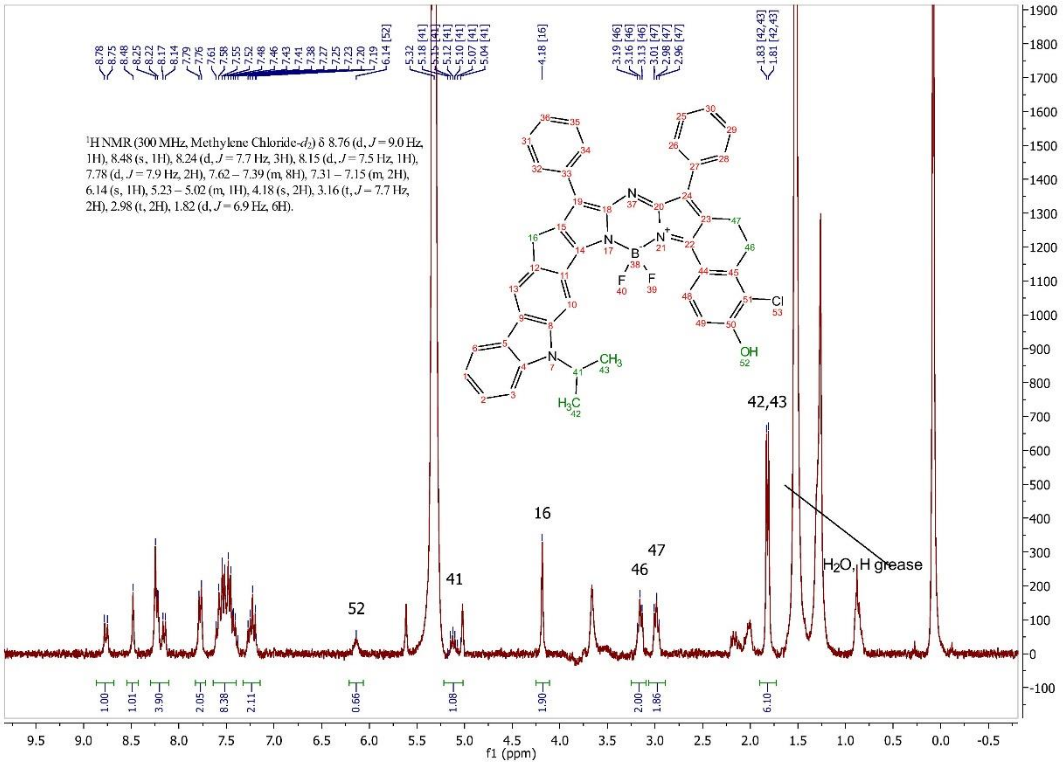

Figure S 19. H NMR (300 MHz, CD_2Cl_2) of **aza-OHCl.**

MS Data

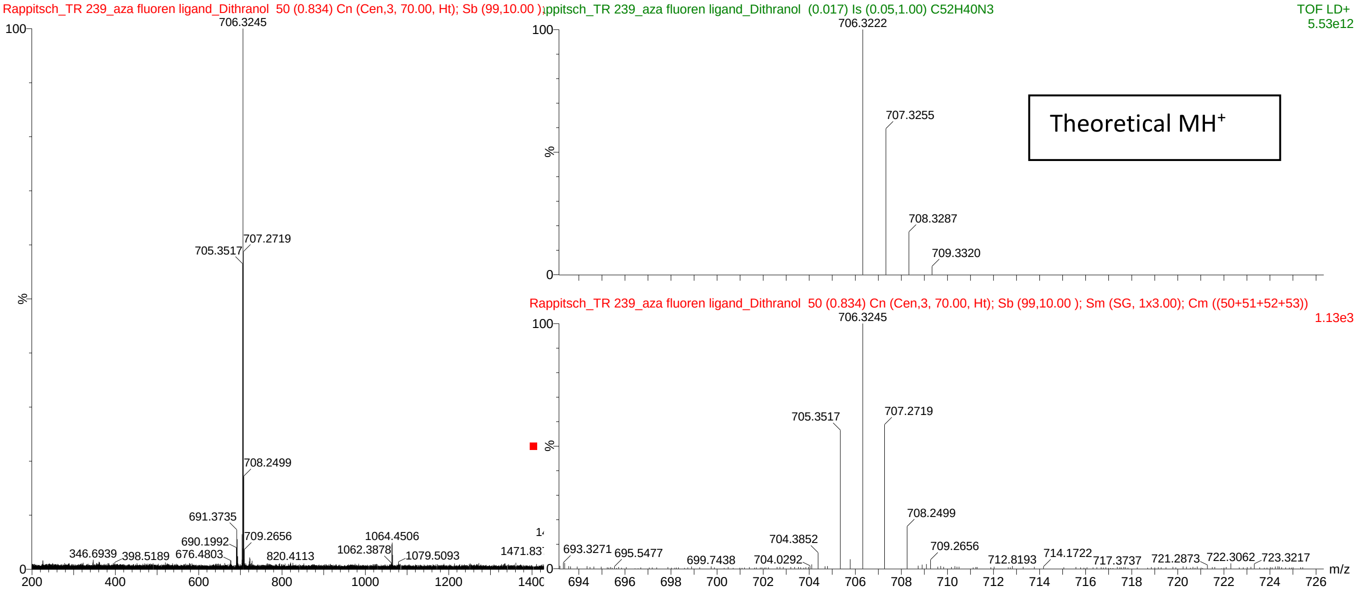

Figure S 20. Mass spectra (MALDI-TOF) of aza-BODIPY ligand **2** in Dithranol matrix.

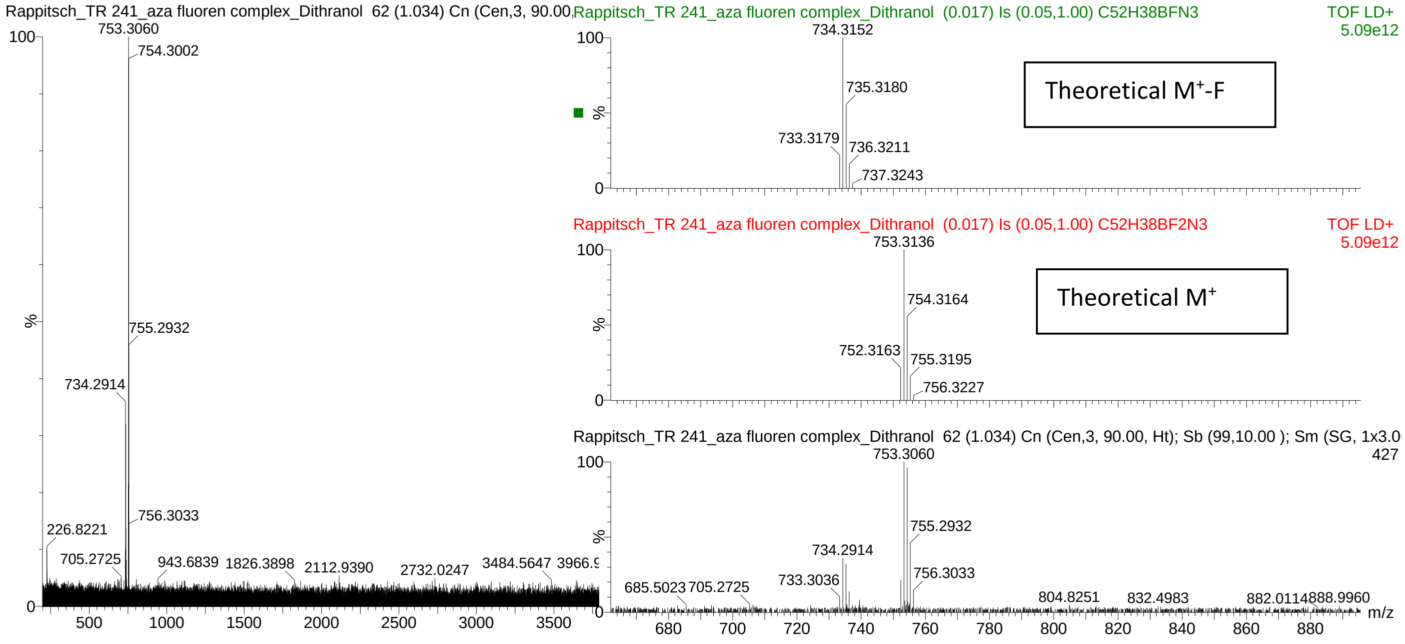

Figure S 21. Mass spectra (MALDI-TOF) of **aza-FL** in Dithranol matrix.

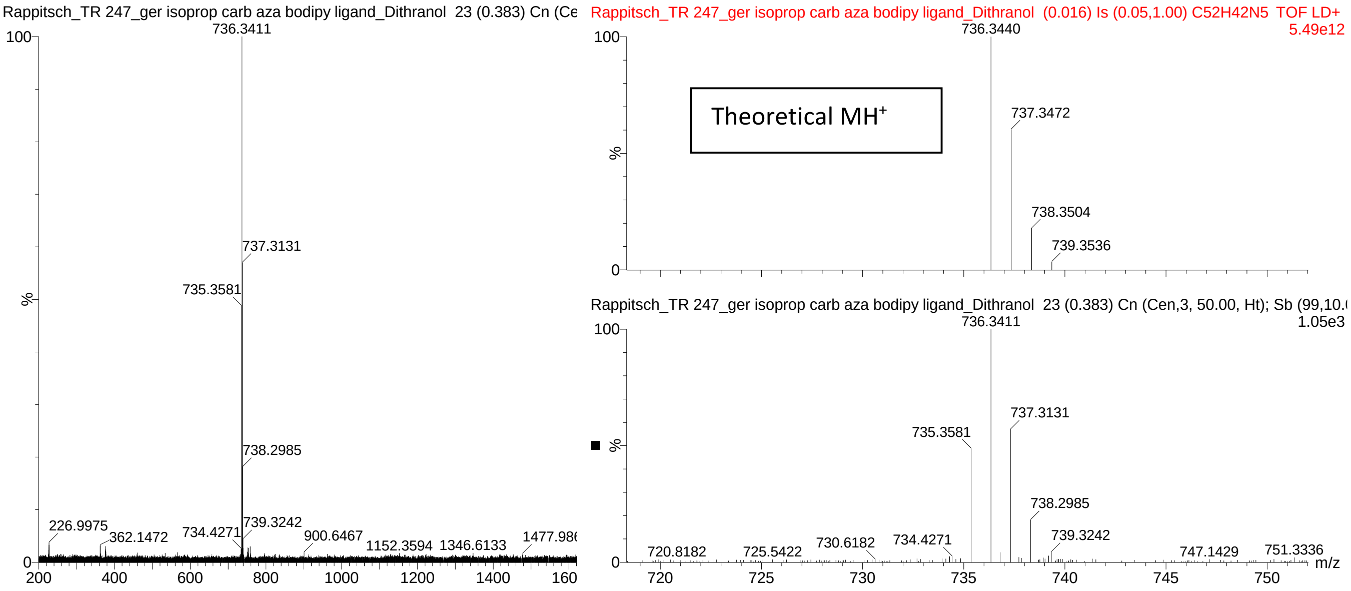

Figure S 22. Mass spectra (MALDI-TOF) of aza-BODIPY ligand **4** in Dithranol matrix.

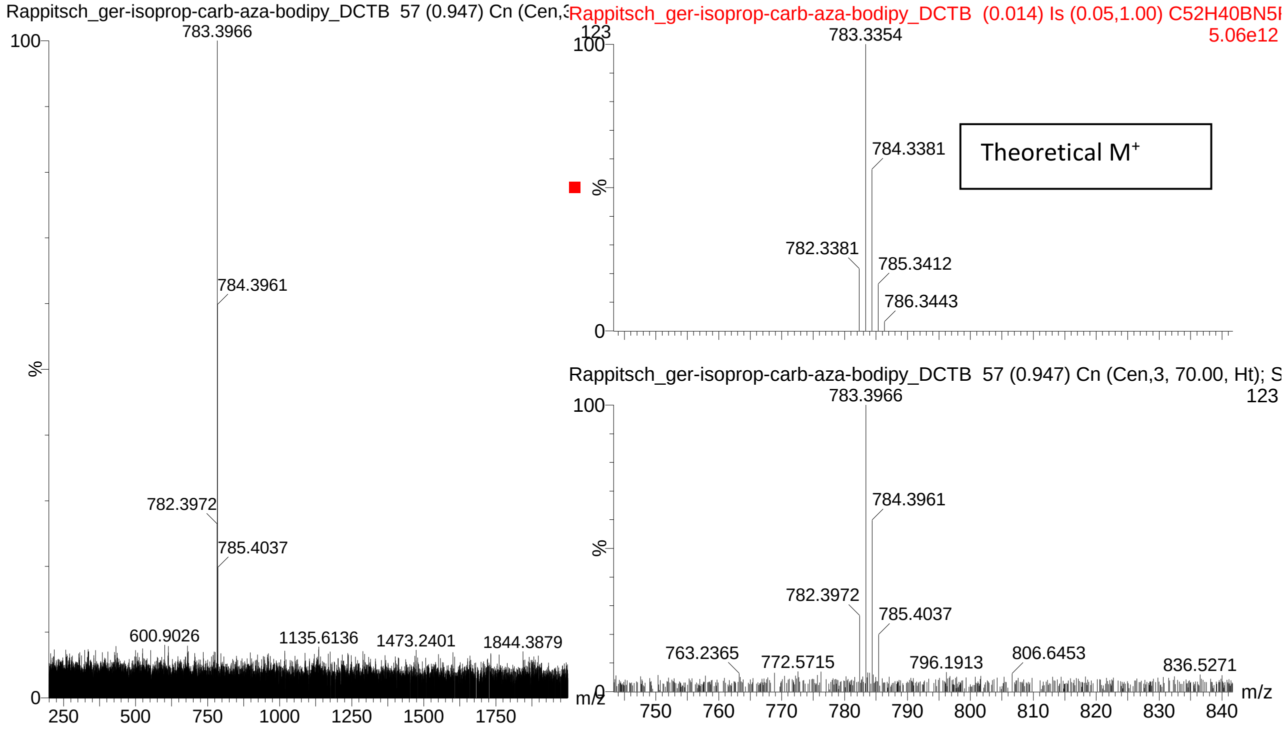

Figure S 23. Mass spectra (MALDI-TOF) of **aza-CZ** in DCTB matrix.

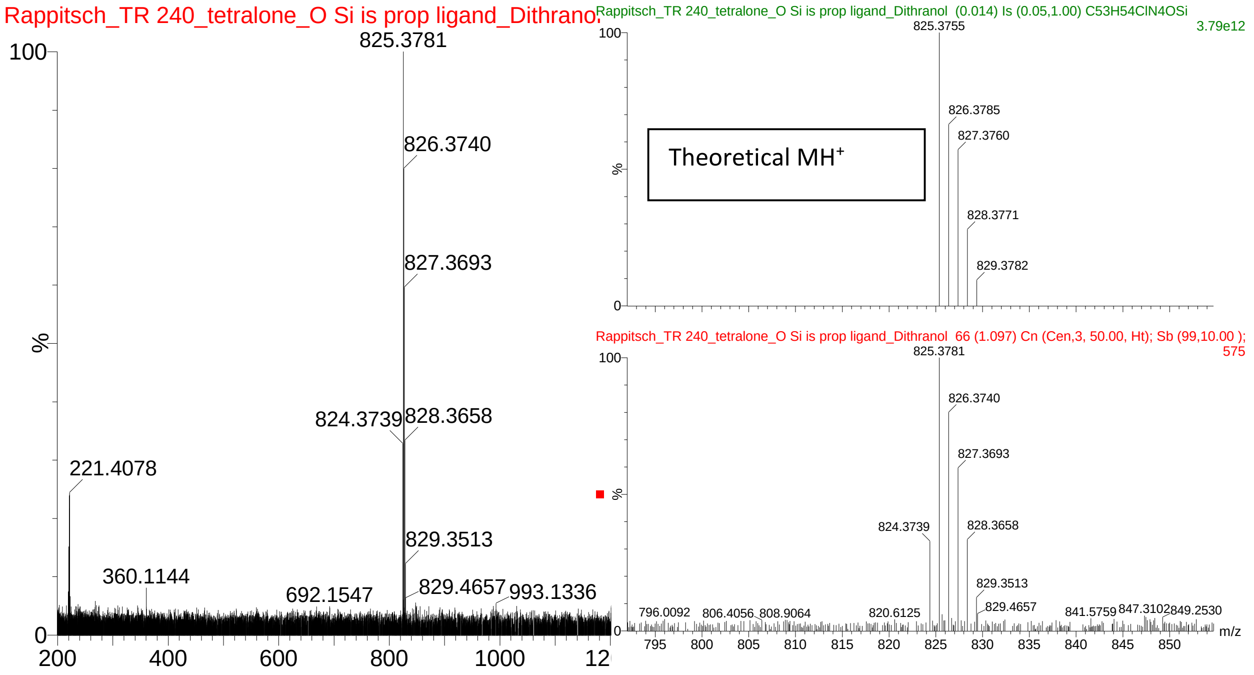

Figure S 24. Mass spectra (MALDI-TOF) of aza-BODIPY ligand **10** in Dithranol matrix.

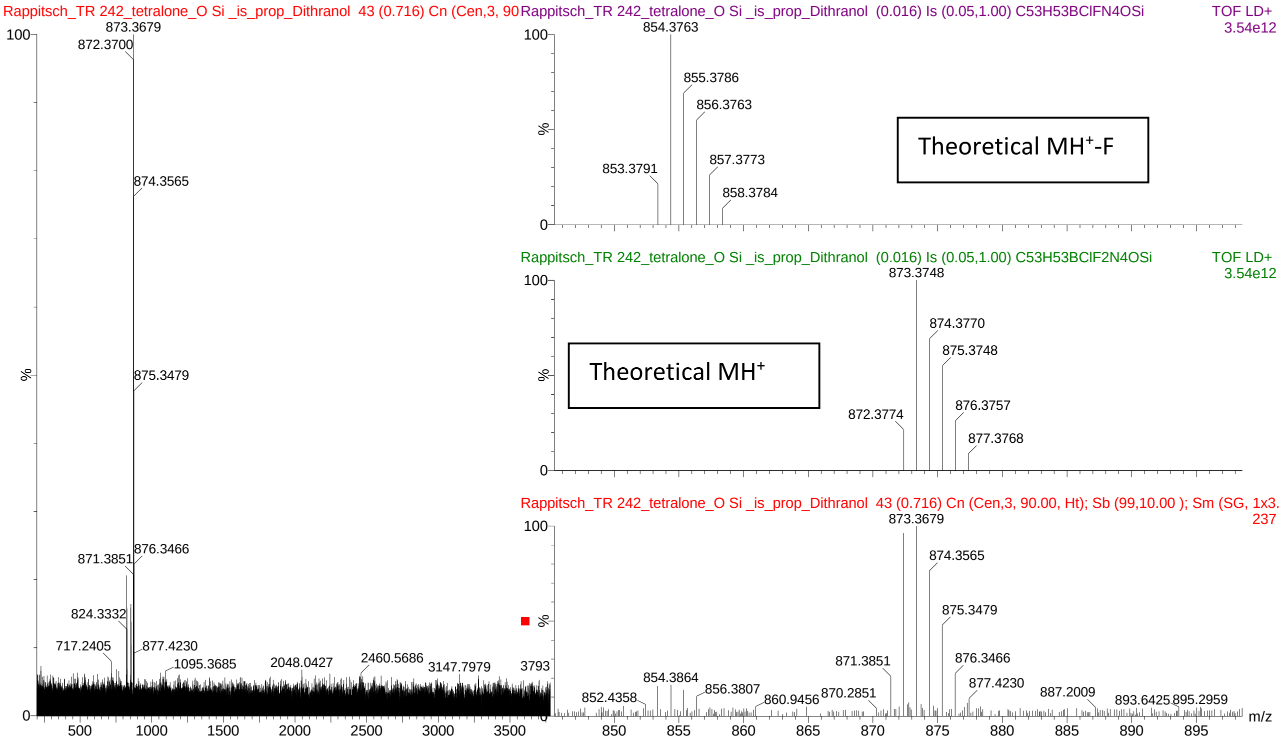

Figure S 25. Mass spectra (MALDI-TOF) of aza-BODIPY complex **11** in Dithranol matrix.

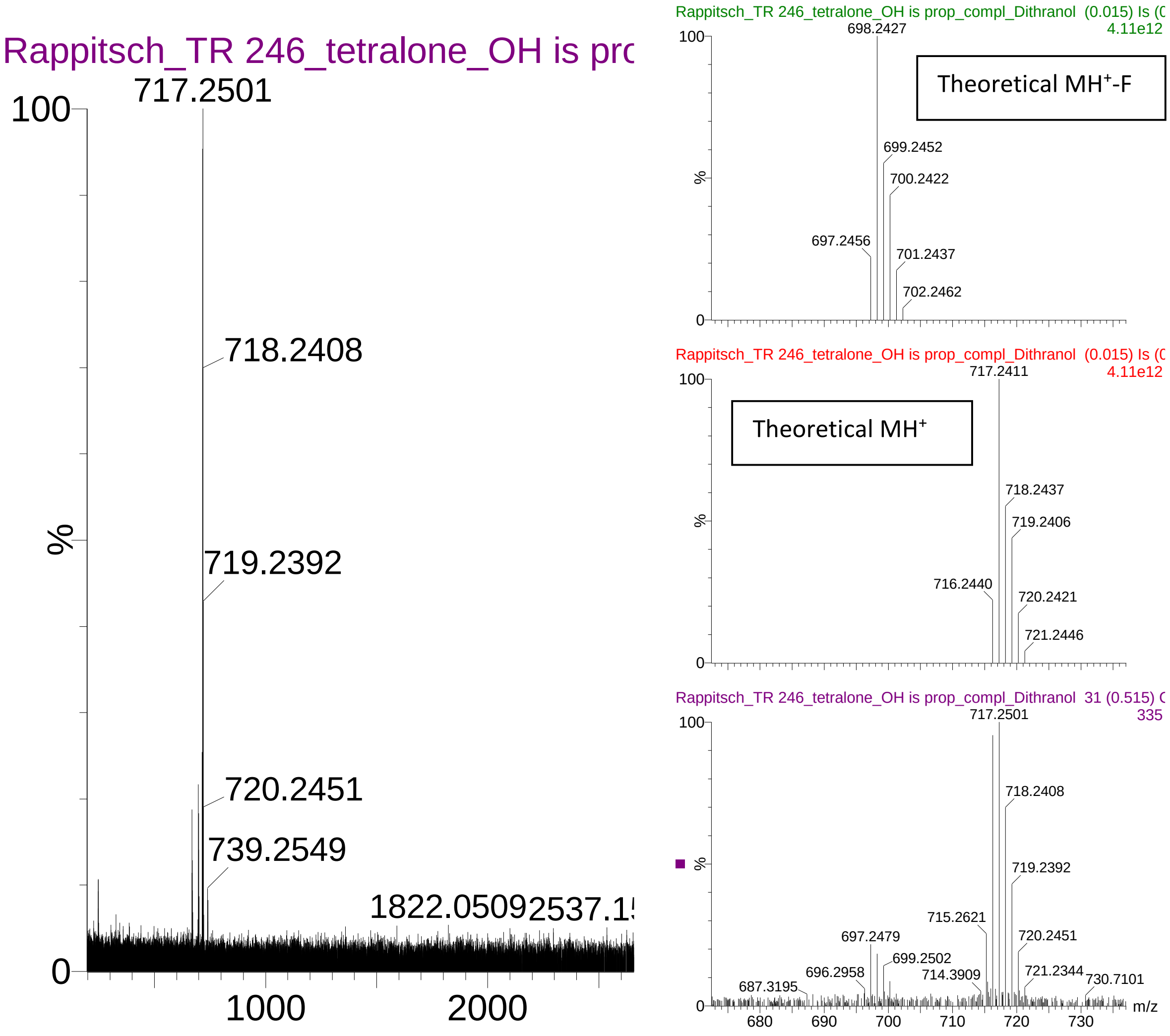

Figure S 26. Mass spectra (MALDI-TOF) of **aza-OHCl** in Dithranol matrix.

References

[1] K. Rurack, M. Spieles, *Anal. Chem.* **2011**, *83*, 1232–1242.
[2] G. M. Fischer, A. P. Ehlers, A. Zumbusch, E. Daltrozzo, *Angew. Chem. Int. Ed.* **2007**, *46*, 3750–3753.
[3] G. M. Fischer, M. K. Klein, E. Daltrozzo, A. Zumbusch, *Eur. J. Org. Chem.* **2011**, *2011*, 3421–3429.
[4] M. Nakamura, H. Tahara, K. Takahashi, T. Nagata, H. Uoyama, D. Kuzuhara, S. Mori, T. Okujima, H. Yamada, H. Uno, *Org. Biomol. Chem.* **2012**, *10*, 6840.
[5] K. Umezawa, D. Citterio, K. Suzuki, *Chem. Lett.* **2007**, *36*, 1424–1425.
[6] Y. Koide, Y. Urano, K. Hanaoka, W. Piao, M. Kusakabe, N. Saito, T. Terai, T. Okabe, T. Nagano, *J. Am. Chem. Soc.* **2012**, *134*, 5029–5031.
[7] J.-Y. Xie, C.-Y. Li, Y.-F. Li, J. Fei, F. Xu, J. Ou-Yang, J. Liu, *Anal. Chem.* **2016**, *88*, 9746–9752.
[8] J. Killoran, L. Allen, J. F. Gallagher, W. M. Gallagher, D. F. O'Shea, *Chem Commun* **2002**, 1862–1863.
[9] J. Killoran, L. Allen, J. F. Gallagher, W. M. Gallagher, D. F. O'Shea, *Chem Commun* **2002**, 1862–1863.
[10] W. Zhao, E. M. Carreira, *Chem. - Eur. J.* **2006**, *12*, 7254–7263.
[11] H. Lu, S. Shimizu, J. Mack, Z. Shen, N. Kobayashi, *Chem. - Asian J.* **2011**, *6*, 1026–1037.
[12] L. Zhang, L. Zhao, K. Wang, J. Jiang, *Dyes Pigments* **2016**, *134*, 427–433.
[13] R. Gresser, M. Hummert, H. Hartmann, K. Leo, M. Riede, *Chem. - Eur. J.* **2011**, *17*, 2939–2947.
[14] H. C. Daly, G. Sampedro, C. Bon, D. Wu, G. Ismail, R. A. Cahill, D. F. O'Shea, *Eur. J. Med. Chem.* **2017**, *135*, 392–400.
[15] X.-D. Jiang, L. Jia, Y. Su, C. Li, C. Sun, L. Xiao, *Tetrahedron* **2019**, *75*, 4556–4560.
[16] X. Zhang, H. Yu, Y. Xiao, *J. Org. Chem.* **2012**, *77*, 669–673.
[17] W. Sheng, Y. Wu, C. Yu, P. Bobadova-Parvanova, E. Hao, L. Jiao, *Org. Lett.* **2018**, *20*, 2620–2623.
[18] W. Sheng, J. Cui, Z. Ruan, L. Yan, Q. Wu, C. Yu, Y. Wei, E. Hao, L. Jiao, *J. Org. Chem.* **2017**, *82*, 10341–10349.
[19] J. Cui, W. Sheng, Q. Wu, C. Yu, E. Hao, P. Bobadova-Parvanova, M. Storer, A. M. Asiri, H. M. Marwani, L. Jiao, *Chem. - Asian J.* **2017**, *12*, 2486–2493.
[20] W. Sheng, Y.-Q. Zheng, Q. Wu, Y. Wu, C. Yu, L. Jiao, E. Hao, J.-Y. Wang, J. Pei, *Org. Lett.* **2017**, *19*, 2893–2896.
[21] L. Jiao, Y. Wu, S. Wang, X. Hu, P. Zhang, C. Yu, K. Cong, Q. Meng, E. Hao, M. G. H. Vicente, *J. Org. Chem.* **2014**, *79*, 1830–1835.
[22] Q. Bellier, S. Pégaz, C. Aronica, B. L. Guennic, C. Andraud, O. Maury, *Org. Lett.* **2011**, *13*, 22–25.
[23] A. Loudet, R. Bandichhor, K. Burgess, A. Palma, S. O. McDonnell, M. J. Hall, D. F. O'Shea, *Org. Lett.* **2008**, *10*, 4771–4774.
[24] Y. Kubo, T. Shimada, K. Maeda, Y. Hashimoto, *New J. Chem.* **2020**, DOI 10.1039/C9NJ04612G.
[25] N. Balsukuri, N. J. Boruah, P. E. Kesavan, I. Gupta, *New J. Chem.* **2018**, *42*, 5875–5888.
[26] X.-D. Jiang, D. Xi, C. Sun, J. Guan, M. He, L.-J. Xiao, *Tetrahedron Lett.* **2015**, *56*, 4868–4870.
[27] H. Yamane, S. Ohtani, K. Tanaka, Y. Chujo, *Tetrahedron Lett.* **2017**, *58*, 2989–2992.
[28] X.-D. Jiang, J. Guan, Q. Li, C. Sun, *Asian J. Org. Chem.* **2016**, *5*, 1063–1067.
[29] P. Majumdar, J. Mack, T. Nyokong, *RSC Adv.* **2015**, *5*, 78253–78258.
[30] L. Jiao, Y. Wu, Y. Ding, S. Wang, P. Zhang, C. Yu, Y. Wei, X. Mu, E. Hao, *Chem. - Asian J.* **2014**, *9*, 805–810.
[31] Y. Wu, C. Cheng, L. Jiao, C. Yu, S. Wang, Y. Wei, X. Mu, E. Hao, *Org. Lett.* **2014**, *16*, 748–751.
[32] E. M. A. Al-Imarah, P. J. Derrick, A. Partridge, *J. Photochem. Photobiol. Chem.* **2017**, *337*, 82–90.
[33] L. Zhu, W. Xie, L. Zhao, Y. Zhang, Z. Chen, *RSC Adv.* **2017**, *7*, 55839–55845.
[34] X.-D. Jiang, D. Xi, B. Le Guennic, J. Guan, D. Jacquemin, J. Guan, L.-J. Xiao, *Tetrahedron* **2015**, *71*, 7676–7680.
[35] X. Jiang, T. Zhang, C. Sun, Y. Meng, L. Xiao, *Chin. Chem. Lett.* **2019**, *30*, 1055–1058.
[36] H. Ş. Çınar, Ş. Özçelik, K. Kaya, Ö. D. Kutlu, A. Erdoğmuş, A. Gül, *J. Mol. Struct.* **2020**, *1200*, 127108.
[37] N. Balsukuri, M. Y. Lone, P. C. Jha, S. Mori, I. Gupta, *Chem. - Asian J.* **2016**, *11*, 1572–1587.
[38] J. Zuo, H. Pan, Y. Zhang, Y. Chen, H. Wang, X.-K. Ren, Z. Chen, *Dyes Pigments* **2020**, *183*, 108714.
[39] Ł. Łapok, I. Cieślar, T. Pędziński, K. M. Stadnicka, M. Nowakowska, *ChemPhysChem* **2020**, *21*, 725–740.
[40] L. Bai, P. Sun, Y. Liu, H. Zhang, W. Hu, W. Zhang, Z. Liu, Q. Fan, L. Li, W. Huang, *Chem. Commun.* **2019**, *55*, 10920–10923.
[41] W. Sheng, F. Chang, Q. Wu, E. Hao, L. Jiao, J.-Y. Wang, J. Pei, *Org. Lett.* **2020**, *22*, 185–189.
